# The PII protein interacts with the Amt ammonium transport and modulates nitrate/nitrite assimilation in mycobacteria

**DOI:** 10.3389/fmicb.2024.1366111

**Published:** 2024-03-25

**Authors:** Delfina Ensinck, Edileusa C. M. Gerhardt, Lara Rollan, Luciano F. Huergo, Hugo Gramajo, Lautaro Diacovich

**Affiliations:** ^1^Instituto de Biología Molecular y Celular de Rosario (IBR-CONICET), Facultad de Ciencias Bioquímicas y Farmacéuticas, Universidad Nacional de Rosario, Rosario, Argentina; ^2^Department of Biochemistry and Molecular Biology, Universidade Federal do Paraná, Curitiba, Paraná, Brazil; ^3^Setor Litoral, Federal University of Paraná, Universidade Federal do Paraná (UFPR), Matinhos, Paraná, Brazil; ^4^Graduated Program in Sciences-Biochemistry, Universidade Federal do Paraná (UFPR), Curitiba, Paraná, Brazil

**Keywords:** mycobacteria, PII protein, nitrogen metabolism regulation, nitrate/nitrite assimilation, ammonium transport

## Abstract

PII proteins are signal transduction proteins that belong to a widely distributed family of proteins involved in the modulation of different metabolisms in bacteria. These proteins are homotrimers carrying a flexible loop, named T-loop, which changes its conformation due to the recognition of diverse key metabolites, ADP, ATP, and 2-oxoglutarate. PII proteins interact with different partners to primarily regulate a set of nitrogen pathways. In some organisms, PII proteins can also control carbon metabolism by interacting with the biotin carboxyl carrier protein (BCCP), a key component of the acetyl-CoA carboxylase (ACC) enzyme complex, inhibiting its activity with the consequent reduction of fatty acid biosynthesis. Most bacteria contain at least two PII proteins, named GlnB and GlnK, with different regulatory roles. In mycobacteria, only one PII protein was identified, and the three-dimensional structure was solved, however, its physiological role is unknown. In this study we purified the *Mycobacterium tuberculosis* (*M. tb*) PII protein, named GlnB, and showed that it weakly interacts with the AccA3 protein, the α subunit shared by the three different, and essential, Acyl-CoA carboxylase complexes (ACCase 4, 5, and 6) present in *M. tb*. A *M. smegmatis* deletion mutant, ∆Ms*PII,* exhibited a growth deficiency on nitrate and nitrite as unique nitrogen sources, and accumulated nitrite in the culture supernatant. In addition, *M. tb* PII protein was able to interact with the C-terminal domain of the ammonium transporter Amt establishing the ancestral role for this PII protein as a GlnK functioning protein.

## Introduction

Nitrogen is a constituent of essential cellular metabolites, such as nucleotides and aminoacids, consequently, all organisms must be provided with this macro-element. Actinomycetes, including *M. smegmatis*, can metabolize different nitrogen sources, and may adapt their metabolism to a number of environmental conditions and situations of nitrogen starvation (Jeßberger et al., 2013). For instance, during the infection process within the eukaryotic host cells, *Mycobacterium tuberculosis* (*M. tb*) is exposed to nutrient starvation, hypoxia, low pH, and reactive species of oxygen and nitrogen; hence, the bacterium must adjust its global metabolism to establish an asymptomatic latent state. Mycobacteria are also capable of exploiting diverse nitrogen sources available within the host; consequently, the regulation of nitrogen metabolism is essential for the virulence and survival of *M. tb* (Gouzy et al., 2014).

In many bacteria and plants, the proteins belonging to the PII superfamily function as sensors and regulators of nitrogen status (Ninfa and Atkinson, 2000; Arcondéguy et al., 2001; Forchhammer et al., 2022). These proteins consist of 12–13 kDa homo-trimers with three exposed T-loops that can be modified allosterically by binding ATP, ADP and 2-oxoglutarate (2-OG) (Macpherson et al., 1998; Xu et al., 1998; Radchenko et al., 2010) or by reversible covalent modifications such as uridylation or adenylation of the conserve Tyrosine-51 residue (Son and Rhee, 1987; Hesketh et al., 2002; Strösser et al., 2004; Williams et al., 2013). These modifications may alter the structure of the T-loops, allowing or constraining the interaction of the PII proteins with different protein partners or targets (Huergo et al., 2013). The genome of *Escherichia coli,* and other bacteria, contains at least two genes that encode for two putative PII proteins, named as GlnB and GlnK, which share approximately 67% of sequence identity at the aminoacids levels. In *E. coli*, GlnB regulates the expression and activity of the glutamine synthetase (GlnA) either through its action on the two-component system (NtrB-NtrC) or by interacting with the adenylyl-transferase GlnE, depending on the levels of glutamine in the cells (Bueno et al., 1985). Recently, GlnB was also reported to be involved in the regulation of carbon metabolism, through its interaction with the biotin carboxyl carrier protein (BCCP) component of the acetyl-CoA carboxylase complex (Feria Bourrellier et al., 2010; Gerhardt et al., 2015; Hauf et al., 2016; Rodrigues et al., 2019) leading to the inhibition of the enzyme activity. On the other hand, *glnK* is arranged in an operon together with the gene of an ammonium transporter, *amtB*. In actinomycetes, this operon also contains the gene encoding for the adenylyl transferase, *glnD* (Thomas et al., 2000; Arcondéguy et al., 2001). The function of GlnK is to bind AmtB, under nitrogen excess conditions, preventing the entry of ammonium into the cell. When the concentration of nitrogen is limited, GlnK binds ATP and 2-OG and later is uridylated by GlnD; this modification impedes the interaction of this protein with the transporter AmtB allowing the influx of ammonium (Coutts and Thomas, 2002; Durand and Merrick, 2006).

Mycobacteria genomes contain a unique gene encoding for PII protein. The *M. tb* PII protein (encoded by Rv2919c) was annotated as GlnB due to the highest sequence similarity (61.6%) with *E. coli* GlnB. In contrast, the genetic environment of the GlnB encoding gene could suggest that this protein may have the role of GlnK, as it is part of the *amt-glnB-glnD* operon, where *amt* is an orthologue of *amtB* (Thomas et al., 2000). In addition, the structure of *M. tb* PII protein also shows that this polypeptide has features of GlnK-like proteins (Shetty et al., 2010). However, the resulting structure of the phylogenetic tree indicates that the PII proteins of actinobacteria would form a different lineage from the GlnK and GlnB of proteobacteria (Sant'Anna et al., 2009; Bandyopadhyay et al., 2010). Interestingly, the gene encoding *M. tb* PII is not essential for growth in rich media, but it is relevant for survival in primary murine macrophages (Rengarajan et al., 2005).

For mycobacteria, the preferred nitrogen sources are ammonium or aminoacids (Amon et al., 2009; Gouzy et al., 2014); however, they also have the capacity to metabolize other inorganics compounds like nitrate or nitrite. The utilization of different nitrogen sources is modulated at the transcriptional level by GlnR. This key regulator control the expression of a number of genes related to nitrogen metabolism in *M. smegmatis*, including ammonium transporters, and is required for *M. tb* to utilize nitrate and nitrite as unique nitrogen source (Amon et al., 2008; Malm et al., 2009; Jenkins et al., 2013; Williams et al., 2015). A second regulatory protein was identified in *M. smegmatis*, NnaR, which cooperates with GlnR to modulate the transcriptional activation of the *nirBD* operon (Antczak et al., 2018). However, several aspects of the control and connections of nitrogen pathways are still unknown in mycobacteria, including the role of PII protein (Gouzy et al., 2014). In this work, we evaluated the functions of the purified PII protein of *M. tb* at the biochemical level adding evidence for the role of this protein as a GlnK-like. Furthermore, we constructed a ∆Ms*PII* deletion mutant strain in *M. smegmatis*, and its physiological characterization suggests a new role for the PII proteins in the assimilation of nitrogen in mycobacteria.

## Experimental procedures

### Bacterial strains, growth and transformation conditions

The bacterial strains and plasmids used in this work are listed in Table 1. The *E. coli* strains were used for cloning proposes and protein heterologous expression. They were transformed according to (Sambrook et al., 1989). Transformants were selected on Luria–Bertani (LB) media at 37°C supplemented with the appropriate antibiotics: 50 μg kanamycin (Km) ml^−1^ and/ or 20 μg chloramphenicol (Cm) ml^−1^. *M. smegmatis* mc^2^155, an electroporation proficient mutant of mc^2^6 (Snapper et al., 1990), was routinely grown at 37°C in Middlebrook 7H9 (BD-Difco) medium supplemented with 0.03% tyloxapol or in solid medium Middlebrook 7H10 (BD-Difco) supplemented with the appropriate antibiotics if required 15 μg kanamycin ml^−1^, 20 μg gentamycin ml^−1^ or 50 μg hygromycin ml^−1^. For nitrogen analysis, bacteria were grown in modified Sauton’s minimal medium (Jenkins et al., 2013) supplemented with different nitrogen sources. Cells were cultivated in 15 mM ammonium sulfate (Sigma) to log phase and washed twice with nitrogen-free medium before inoculating fresh medium with the required nitrogen source.

**Table 1:** Bacteria strains and plasmids.

**Bacteria strain/plasmids**	**Genotype**	**Source/reference**
***E. coli***		
DH5α	F^−^ Φ80*lacZ*∆MSM15 ∆MS (*lacZYA*-*argF*) U169 *endA1 recA1 hsdR17 deoR supE*44 *thi-1 gyrA96 relA1*	Hanahan, 1983
BL12 (DE3)	F^−^ *ompT* r_B_^−^ m_B_^−^ (DE3)	Studier and Moffatt, 1986
***M**.**smegmatis***		
mc^2^155	*M. smegmatis* electroporation-efficient mutant strain of mc26	Snapper et al., 1990
∆Ms*PII*	*M. smegmatis* mc2155 background. Deletion of *MSMEG_2426* gene	This study
**Plasmids**		
pJET1.2	Used for cloning PCR products	Thermo Fisher Scientific
pET28a(+)	Km^r^, Expression vector T7 promoter	Novagen
pMVHG1	Hyg^r^, Expression vector Hsp60 promoter for mycobacteria	Grzegorzewicz et al., 2012
pPR27	Gm^r^, *sacB, xylE.* Vector with a thermo-sensitive *ori* for mycobacteria that promotes homologous recombination	Pelicic et al., 1997
pMtGlnK	Km^r^ (pET29a). Expresses *M. tb* GlnB	Gerhardt et al., 2017
pLH25PET	Km^r^ (pET28a). Expresses *A. brasiliense* GlnB	Huergo et al., 2005
pTRPETGlnB	Km^r^ (pET29a). Expresses *E. coli* GlnB	Gerhardt et al., 2015
pHA3	Km^r^ (pET28a). Expresses *M. tb* AccA3 His tag fusion	Gago et al., 2006
pD5	Km^r^ (pET28a). Expresses *M. tb Mtub* AccD5 His tag fusion	Gago et al., 2006
pAccE5	Km^r^ (pET28a). Expresses *M. tb* AccE5 His tag fusion	Gago et al., 2006
pD6MT	Km^r^ (pET24b). Expresses *M. tb* AccD6 His tag fusion	Kurth et al., 2009
pCY216	Cm^r^. Vector expressing *E. coli birA* gene	Chapman-Smith et al., 1994
pDE2	Km^r^(pET24). Expresses *M. tb* PII His tag fusion	This study
pDE13	Vector for ∆Ms*PII* mutant construction, *sacB*, *xylE*, GmR	This study
pDE35	Hyg^r^ (pMEVGH1). Expresses *M. smegmatis* PII	This study
pET2832	Km^r^. Vector for protein expression as a N-terminal His and Thiorredoxin A fusions, with TEV site, cleavable by TEV protease, under the control of T7 promoter.	This study
pNZn2	Km^r^ (pET2832) Expresses the C-terminal domain of *M. tuberculosis* Amt fused to His tagged-Thioredoxin A (TrxA) at N- terminal	This study

### Cloning and molecular biology methods

Isolation of plasmid DNA, restriction enzyme digestion and agarose gel electrophoresis were carried out by conventional methods (Sambrook et al., 1989). The genomic DNA of *M. smegmatis* was obtained as described by Connell (1994).

#### pDE2

The oligonucleotides oDE01 (5’-CGTATCATATG**GAAAACCTG TACTTCCAGGGT**ATGAAGCTGAT CACTGCGA), used to introduce a *Nde*I site (underlined) and TEV site (bold) upstream the *M. tuberculosis glnB* gene, and oDE02 (5′- AGCACAGAAGCTTTA GTTTCATAACGCGTCGTGTC), which introduces a *Hin*dIII site (underlined) at the end of the open reading frame (ORF), were used to amplify the complete *glnB* gene. The PCR product was digested with *Nde*I and *Hind*III and cloned in *Nde*I/*Hind*III-cleaved pET24a(+), yielding pDE2.

#### pDE13

For the construction of the *M. smegmatis* ∆Ms*PII* mutant strain, the upstream region of *MSMEG_2426* gene was amplified with the primers oDE09 (5’-ACTAGTCGGTCTGGGTGAGGG)- and oDE10 (5’-GCTAGCCGTCTTGACATCTTC) introducing *Spe*I and *Nhe*I sites at its extremes. The 1761 bp PCR product was cloned into pJET 1.2 Blunt (Thermo Fisher Scientific) yielding pDE8 plasmid. The downstream region of *MSMEG_2426* gene was amplified with the primers oDE11 (5′- GCTAGCATCGGTGACGGCAAGGTG) and oDE12 (5’-ACTAGTGAGCACGTCGAGCAGTTCC) introducing *Nhe*I and *Spe*I sites at its extremes. The 1842 bp PCR product was cloned into pJET 1.2 Blunt (Thermo Fisher Scientific) yielding plasmid pDE9. Plasmids pDE8 and pDE9 were digested with *Spe*I and *Nhe*I, and the fragments obtained were cloned into *SpeI-*cleaved pPR27 plasmid, yielding pDE13.

#### pDE35

The oligonucleotides oDE13 (5’-CATATGAAGCTGATTACTG CGAT), used to introduce a *Nde*I site (underlined) at the translational start codon of the *M. smegmatis MSMEG_2426* gene, and oDE14 (5’-GAATTCTACAGGGCGTC GGCT), which introduces an *Eco*RI site (underlined) at the end of the open reading frame (ORF), were used to amplify the complete *MSMEG_2426 gene.* The PCR product was digested with *Nde*I and *Eco*RI and cloned in *Nde*I/*Eco*RI-cleaved pMVHG, yielding pDE35.

#### pET2832

Using the vector pET32a(+) as a template and the primers 32AseIup (5′- GAGATATATTAATATGAGCGATAAAATTATTC), which introduces an *Ase*I site (underlined), and 32TEVdn (5′- GGTG CATATG**ACCCTGGAAGTACAGGTTTTC**GCCAGAACCAGAAC CGGCC) introducing *Nde*I (underlined) and TEV (bold) sites, a fragment of 384 pb was amplified. This PCR product was cloned into the pET28a(+) vector digested with *Nde*I, yielding pET2832. This vector allows the expression of proteins as His and TrxA tags fusions, with a TEV digestion site.

#### pNZn2

The oligonucleotides oDE27 (5′- CCTGTA CTTCCAGGGTCATA TGATCGGGCTCAGGC) and oDE28 (5′- GCTCGAGTGCGGC CGCAAGCT TTCATTTCGGCTC) were used to amplify the last 183 pb (61 codons) of the *Rv2920c* gene (*amt*_CTR_). The PCR product was cloned in *Nde*I/*Hin*dIII-cleaved pET2832 by AQUA Cloning (Beyer et al., 2015), yielding pNZn2.

### Expression, purification and protein methods

Recombinant *M. tuberculosis* His-tagged ACCase subunits AccA3, AccD5, AccD6, and AccE5 were purified as described in Gago et al. (2006). The same protocol was applied for His-tagged *M. tb* PII and Amt_CTR_ fused to TrxA. After dialysis, TrxA was removed through digestion with TEV protease. In brief, the Amt_CTR_-TrxA fraction was incubated with TEV protease (30:1) for 2 h at room temperature, followed by an additional 2 h at 4°C. The mixture was then loaded onto a Ni-NTA column, and cleaved Amt_CTR_ was subsequently recovered. PII proteins from *E. coli, A. brasilense* and *M. tb* were obtained without tags, according to Gerhardt et al. (2015, 2017).

Proteins were analyzed by SDS-PAGE (Laemmli, 1970). Protein contents were determined by measuring A280 nm to the solutions and by the Bradford method, using BSA as standard.

For the production of polyclonal antibodies anti PII, a rabbit was immunized biweekly with purified *M. tb* PII, emulsified in Freund’s complete adjuvant (Sigma) at a 1:1 (v/v) ratio. Antisera against PII were elicited in rabbit following conventional procedures (Burnette, 1981, Anal. Biochem). The anti-*M. tb* PII was used to evaluate the presence of *M. tb* or *M. smegmatis* PII proteins (88.4% identity), and both proteins were recognized by the antibody (Supplementary Figure S1A).

PII from mycobacteria were detected by Western blot analysis. After electrophoretic separation, proteins were transferred onto nitrocellulose membranes (Bio-Rad). Anti-*M. tb* PII was used at a 1:300 dilution, and anti-RpoB at 1:10,000 dilution, as a control. Antigenic polypeptides were visualized using an alkaline phosphatase-tagged secondary antibody. For biotinylated proteins western blotting procedure was modified as described by Nikolau et al. (1985). Proteins were probed alkaline phosphatase-streptavidin conjugate (AP-streptavidin diluted 1:10,000) (Bio-Rad).

### *In vivo* reversible adenylation of PII

*M. smegmatis* mc^2^155 was grown in 0.5 mM (NH_4_)_2_SO_4_ to an OD 600 nm 0.6–0.8 and half of the culture was subjected to a shock of 15 mM of ammonium sulfate, for 15 min (Atkinson et al., 1994; Durand and Merrick, 2006; Moure et al., 2012; Rodrigues et al., 2014). The adenylation state of PII in cell-free extracts was determined by electrophoresis on SDS- PAGE or native PAGE and followed by Western blotting as described in (Bonatto et al., 2007).

### Pull down assays

Complex formation between *M. tb* His-AccA3 and PII without a tag were assessed by pull down as described by Rajendran et al. (2011), with modifications. Pull down assay was performed using 15 μL of magnetic beads (Promega) with interaction buffer (Tris–HCl 50 mM pH 8.0, NaCl 100 mM, glycerol 5%, LDAO 0,01%, imidazole 20 mM; MgCl_2_ 5 mM) and saturating ATP (3.5 mM). Fifteen micrograms of purified His-AccA3 were mixed with 20 μg of PII protein from *M. tb* (Mt), *A. brasilense* (Ab) or *E. coli* (Ec). Proteins eluted from the Ni^2+^ were analyzed by SDS-PAGE and the gel was stained with Coomassie Blue.

The interaction between PII and Amt_CTR_ was analyzed *in vitro*. 10 μg of cell extracts expressing His-tagged *M. tb* PII were mixed with 10 μg of purified Amt_CTR_ without tag. The mixture was prepared in the presence of either 1 mM ATP or 1 mM ATP along with 1.5 mM 2-OG. After incubating the mixture for 1 h at 4°C, it was loaded into a Ni^2+^-NTA column that had been pre-equilibrated with PBS containing ATP, or ATP and 2-OG. The column was then washed with PBS, supplemented with the ATP and 2-OG, using 10 column volumes. Finally, the bound proteins were eluted from the column using PBS containing 250 mM imidazole. SDS-PAGE was performed to analyze the samples obtained from the column.

### Acyl-CoA carboxylase activity

ACCase activities in cell-free extracts and *in vitro* reconstituted complexes were determined by following the incorporation of radioactive HCO_3_^−^ into acid non-volatile material, as previously described (Diacovich et al., 2002). Substrate concentrations were 0.5 mM for acetyl-CoA or propionyl-CoA. One unit of enzyme activity catalyzed the incorporation of 1 mmol ^14^C into acid-stable products min^−1^.

### Construction of PII mutant

For the construction of the *M. smegmatis* mutant ∆Ms*PII* the plasmid pDE13 was used to transform *M. smegmatis* cells. The mutant strain generated through a double homologous recombination event was selected according to (Crotta Asis et al., 2021).

### Determination of nitrite concentration

Nitrite concentration was determined using the Griess reaction (Peter, 1879). For the uptake assay, exponentially growing cells in modified Sauton’s minimal medium supplemented with 1 mM nitrite at an OD 600 nm of 0.6–0.8 were harvested by centrifugation at 3,000 × *g* for 10 min at 4° C. Afterward, they were washed twice using modified Sauton’s medium that lacked a nitrogen source. The cultures were then resuspended in fresh medium at pH 7.2 or 9 containing 0.2 mM sodium nitrite and 1 mM ammonium sulfate when indicated, and adjusting to an OD of 600 nm of 0.75. Cells were cultivated under constant shaking of 180 rpm at 37° C. At regular intervals of 15 min, aliquots of the cultures were sampled and centrifuged at 5,000 × *g* for 10 min to remove the cells previous to nitrite quantification.

### Determination of nitrate reductase activity

Crude extracts were obtained from cultures grown in modified Sauton’s minimal medium with 1 mM nitrate. Appropriate amounts of extracts were incubated for 30 min at 30°C in a buffer composed of 0.1 M potassium phosphate (pH 7.5), 0.4 mM NADPH, 0.025 mM FAD, 0.025 mM FMN, and 1 mM NaNO_3_. To measure the concentration of accumulated nitrite, the Griess Reaction was performed (Peter, 1879).

### LC–MS/MS analysis

Cell-free extracts excised from Coomassie-stained SDS-PAGE gels were subjected to digestion with trypsin. Peptide separations were carried out on a nanoHPLC Ultimate3000 (Thermo Scientific) using a nano column EASY-Spray ES903 (50 cm × 50 μm ID, PepMap RSLC C18). The mobile phase flow rate was 400 nl/min, using 0.1% formic acid in water (solvent A) and 0.1% formic acid and 100% acetonitrile (solvent B). The gradient profile was set as follows: 4–35% solvent B for 30 min, 35–90% solvent B for 1 min and 90% solvent B for 5 min. Three microliters of each sample were injected. MS analysis was performed by using a Q-Exactive HF mass spectrometer (Thermo Scientific). For ionization, 1.9 kV of liquid junction voltage and 250°C capillary temperature was used. The full scan method employed a m/z 375–1,600 mass selection, an Orbitrap resolution of 120,000 (at m/z 200), a target automatic gain control (AGC) value of 3e6, and maximum injection times of 100 ms. After the survey scan, the 7 most intense precursor ions were selected for MS/MS fragmentation. Fragmentation was performed with a normalized collision energy of 27 eV and MS/MS scans were acquired with a dynamic first mass. AGC target was 5e^5^, resolution of 30,000 (at m/z 200), intensity threshold of 4.0e4, isolation window of 1.4 m/z units and maximum IT was 200 ms. Charge state screening was enabled to reject unassigned, singly charged, and equal or more than seven protonated ions. A dynamic exclusion time of 15 s was used to discriminate against previously selected ions.

MS data were analyzed with MaxQuant (V: 2.1.4.00) using standardized workflows. Mass spectra *.raw files were searched against a database from *Mycobacterium smegmatis mc2155* (reviewed and unreviewed proteins), entry UP00000075 from *Uniprot*. The “MSMEG_4206” sequence was added to the database. Precursor and fragment mass tolerance were set to 10 ppm and 0.02 Da, respectively, allowing 2 missed cleavages. Fixed modification: carbamidomethylation of cysteines. Variable modifications: protein N-terminal acetylation and methionine oxidation. The statistical analysis was performed with Perseus v1.6.15.0 software (MaxQuant).

### Cellular fractionation

The culture was grown in a modified Sautons’ medium supplemented with 0.5 mM (NH_4_)_2_SO_4_ until reaching an OD of 0.8 at 600 nm. The culture was then split into four fractions. In three of the cultures, (NH_4_)_2_SO_4_ was added to a final concentration of 15 mM, inducing an ammonium shock. After 15 min, the cultures were harvested by centrifugation at 3,000 x g for 10 min at 4°C. The cells were washed twice with PBS and resuspended in 100 μl of PBS containing 1 mM PMSF and either 0.6 mM ADP, or 4.5 mM ATP, and 1.5 mM 2-OG. Then, cultures were lysed using a water bath sonicator (Diagenode), and the lysates were clarified by centrifugation at 11,180 × *g* for 30 min at 4°C. The resulting cell-free extracts were further centrifuged at 16,000 x g for 1 h at 4°C, and the supernatant, representing the cytosolic fraction, was collected. The pellet, containing the membrane fraction, was washed once with PBS, containing the corresponding additives, and centrifuged again for 30 min at 16,000 g. The membranes were then resuspended in 50 μl of PBS. The samples were analyzed by SDS-PAGE followed by western blotting, performed to detect PII and RpoB, serving as a control.

## Results

### Post-translational modification of endogenous PII protein levels in *Mycobacterium smegmatis*

**Figure 1:**
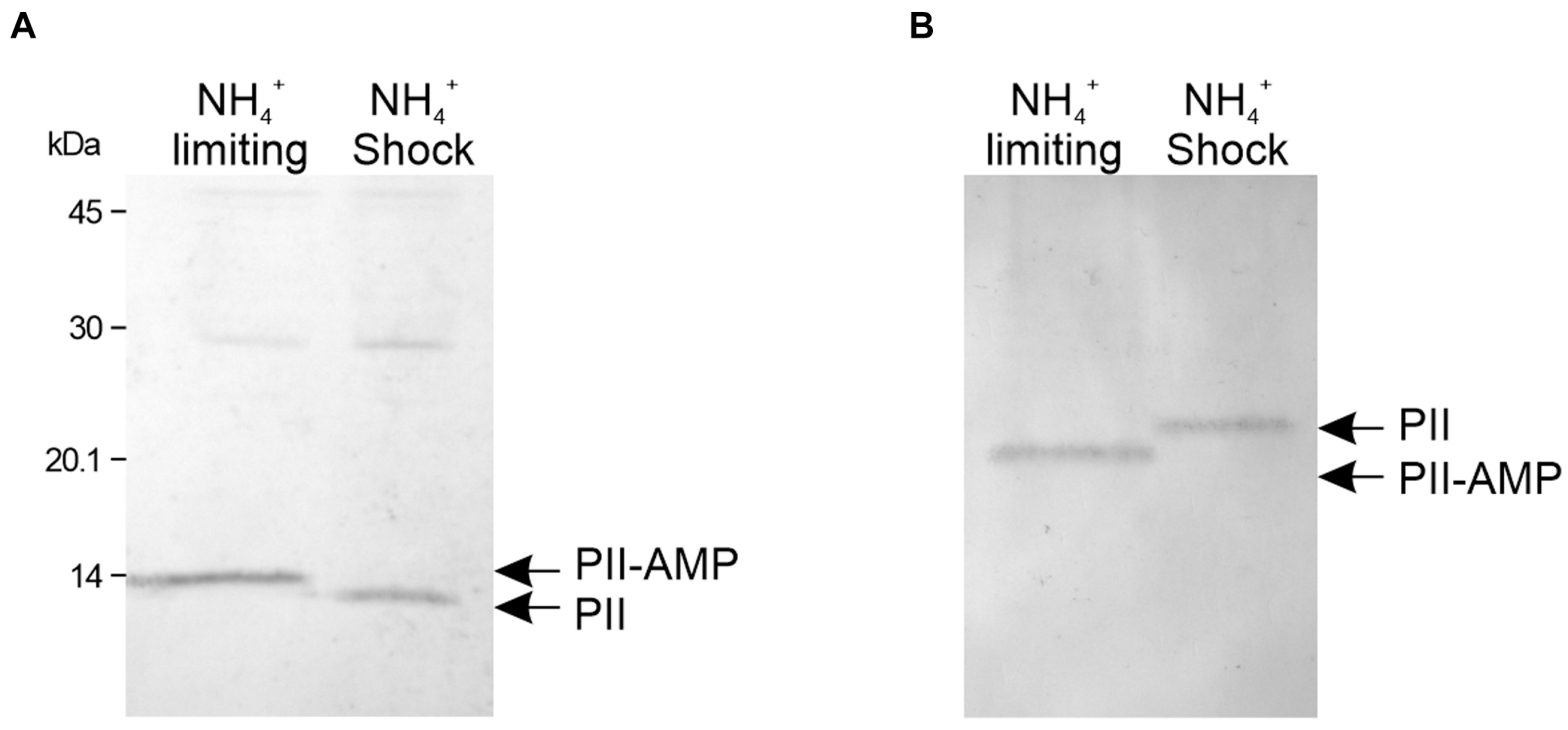
Determination of the adenylation state of PII from *M. smegmatis*. Bacteria were grown in nitrogen-limiting conditions with 0.5 mM of (NH_4_)_2_SO_4_. An aliquot of the culture was subject to an ammonium shock. The mobility of PII was analyzed before (NH_4_^+^ limiting) and after 15 min of ammonium shock (NH_4_^+^ shock). Cell-free extracts were separated by SDS-PAGE **(A)** or native PAGE **(B)** and analyzed by western blot, using a polyclonal anti *M. tb* PII antibody.

The genomes of *M. tb* and *M. smegmatis* contain one type of PII protein-encoding gene. The PII protein expression in mycobacteria is induced in response to nitrogen limitation (Amon et al., 2008; Williams et al., 2013) and PII is rapidly adenylated by GlnD (Williams et al., 2013). To understand the dynamics of PII protein adenylation in response to the nutritional status, we analyzed the state of PII adenylation under different nitrogen regimen. *M. smegmatis* mc^2^155 was grown in modified Sauton’s minimal medium supplemented with 0.5 mM ammonium sulfate (Williams et al., 2013, 2015) to an OD 600 nm of 0.7; at this stage, PII expression can be detected by Western-blot (Supplementary Figure S1A). At this particular OD, the culture was divided into two fractions and one was subjected to a concentration of 30 mM of ammonium, which is considered an ammonium shock (Coutts and Thomas, 2002) After 15 min, cells were collected and protein extracts were analyzed by SDS-PAGE or native gels, and detected by Western blotting. As can be observed in Figures 1A,B, the migration pattern of PII changed after the ammonium shock, with a pattern consistent with the loss of the nucleoside (Atkinson et al., 1994; Durand and Merrick, 2006; Moure et al., 2012; Rodrigues et al., 2014). In native gels, this modification provokes PII to run faster than unmodified PII, due to PII-AMP’s higher negative charge imparted by the AMP residues. On the contrary, in SDS-PAGE, as the gel in Figure 1A, the adenylation of PII produces a delay in protein migration. The impact of adenylation on protein electrophoretic mobility, under denaturing conditions, was validated by LC-MS/MS (data not shown). These results suggest that the adenylation of PII is readily reversible by increasing the availability of ammonium.

### *Mycobacterium tuberculosis* PII interacts with AccA3 but does not modulate Acyl-CoA carboxylase activities

In *E. coli* two PII proteins were described, namely GlnB and GlnK. Both proteins can interact with the BCCP subunit of the acetyl-CoA carboxylase (ACC) complex, but only GlnB is able to interact with the complex inhibiting the ACC holoenzyme activity (Gerhardt et al., 2015). Given that the regulation of ACC by PII is conserved in distantly related organisms, ranging from plants to bacteria (Forchhammer et al., 2022), we hypothesized that such PII function could be also conserved in mycobacteria. In *M. tb* the acetyl-CoA carboxylase activity is present in two enzyme complexes, named acyl-CoA carboxylases (ACCases) based on their relaxed substrate specificity. These two complexes, ACCase 5 and ACCase 6, consist of two subunits: an α-subunit named AccA3, which is shared by both ACCases, containing the biotin carboxylase (BC) and the biotin carboxyl carrier protein (BCCP) domains, and a specific β-subunit carrying the carboxyltransferase (CT) domain, named AccD5 or AccD6, respectively (Gago et al., 2006; Daniel et al., 2007; Kurth et al., 2009; Pawelczyk et al., 2011; Bazet Lyonnet et al., 2014). A third subunit, named ε, is also required for maximal activity of the ACCase 5 complex. These two enzymes are able to carboxylate both acetyl-CoA (ACC activity) and propionyl-CoA (PCC activity), but with different substrate preferences (Gago et al., 2006; Daniel et al., 2007). On the other hand, in *E. coli* there is only one type of ACC enzyme of which the BC and BCCP components are encoded by separated polypeptides.

**Figure 2:**
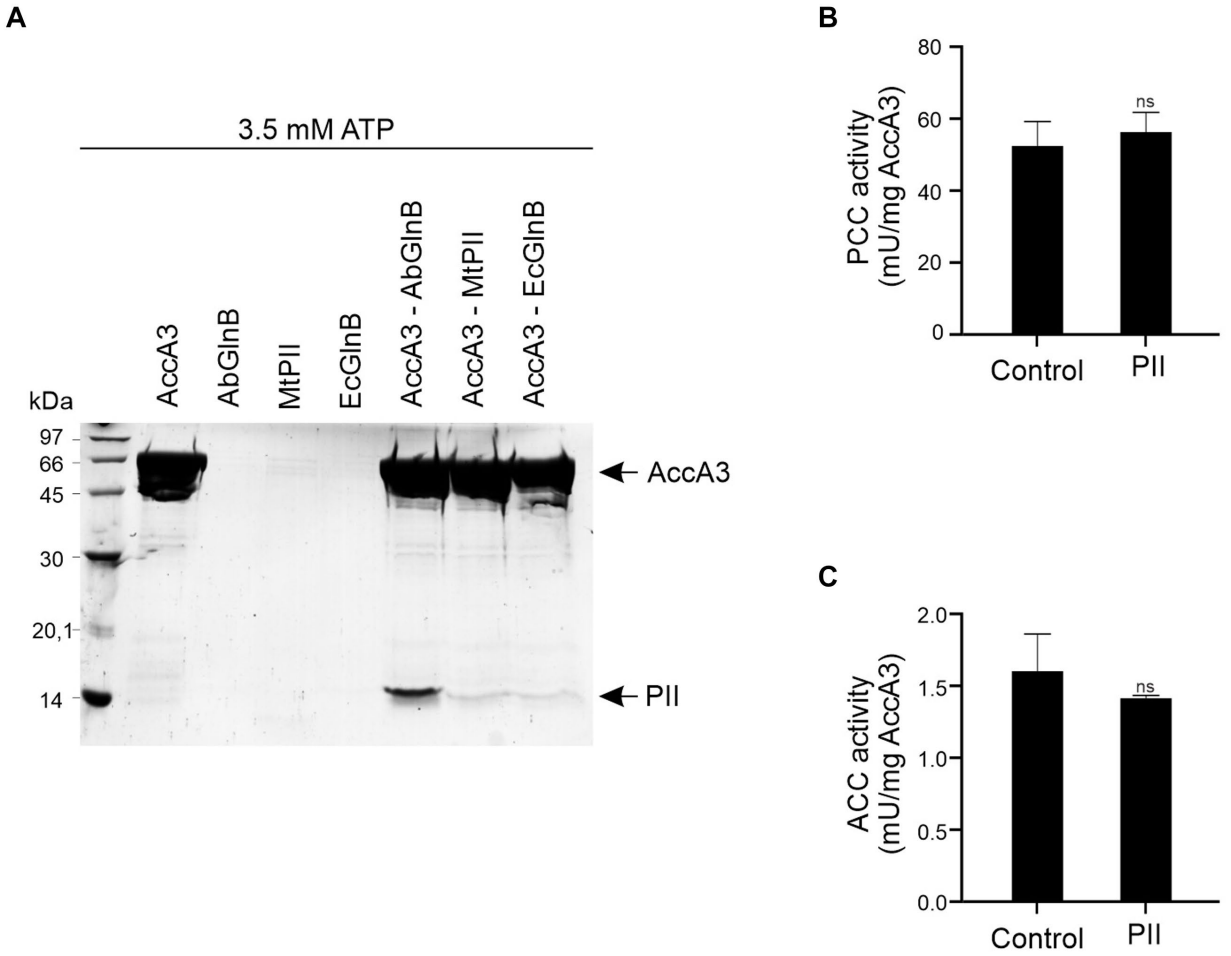
Interaction between PII and AccA3 and influence on ACCasa activities. **(A)** Complex formation between *M. tb* His-AccA3 and PII proteins was assessed by pull-down under saturating concentrations of ATP (3.5 mM), and MgCl_2_ (5 mM). Purified His-AccA3 was mixed with PII protein (without tags) from *M. tb* (Mt), *A. brasilense* (Ab), or *E. coli* (Ec). Proteins eluted from the Ni^2+^ resins were analyzed by SDS-PAGE and the gel was stained with Coomassie Blue. The effect of *M. tb* PII on PCC **(B)** and ACC **(C)** activities of the *in vitro* reconstituted complexes ACCase 5 and ACCase 6, respectively, was measured by determining the amount of radiolabeled HCO_3_^−^ incorporated into malonyl-CoA or methylmalonyl-CoA, in presence or absence of *M. tb* PII. PII was added at a concentration equal to that of the AccA3 subunit (0.8 μM for ACCase 5 and 1.9 μM for ACCase 6). The specific activity is plotted as nmols of malonyl- or methylmalonyl-CoA produced per min, per mg of the AccA3 subunit. Data is reported as the average of quadruplicate and standard deviation. Statistical analysis was performed with a *t*-test at *p* < 0.05, ns, no significant.

To investigate the interaction between PII and the biotinylated subunit of the ACCase, purified AccA3-His_6_ (α-subunit) was immobilized into nickel beads and used as bait which was challenged with purified *M. tb* PII without tags (Gerhardt et al., 2015). Orthologue PII proteins, GlnB from *Azospirillum brasiliense* (Ab) and GlnB from *E. coli* (Ec), which share 60.7 and 61.6% similarity with *M. tb* PII (Mt), respectively, were used as controls. The pull-down assay data revealed that AccA3 interacted strongly with Ab-GlnB but weakly with Ec-GlnB or Mt-PII (Figure 2A). We also observed that the specificity of the AccA3 PII proteins interaction is dependent on the PII effectors. The interaction between AccA3 and PII is only effective in the presence of ATP, but it does not occur in the presence of ADP (Figure 2A and Supplementary Figure S2A). On the other hand, we also used lysozyme as a non-specific protein and showed that this protein does not interact with AccA3, in the same conditions used to detect AccA3-PII interaction (Supplementary Figure S2B).

In order to analyze if these interactions could affect *M. tb* ACCase activities, we assayed the propionyl-CoA carboxylase (PCC) activity *in vitro* using the reconstituted ACCase 5 complex which is composed of AccA3 (BC-BCCP α-subunit), AccD5 (CT), and AccE5 (ε subunit). When Ab-GlnB PII protein was incorporated into the reaction mix, an inhibition of the PCC activity was detected (Supplementary Figure S3), which is consistent with the strong interaction observed in the pull-down assay (Figure 2A). This data support that the interaction between ACCase and PII acts to reduce ACC/PCC activity as described previously in bacteria such as *E. coli* and *Streptomyces hygroscopicus* var. *ascomyceticus* (Wang et al., 2021). However, in the presence of *M. tb* PII no significant modulation of PCC activity was detected (Figure 2B), which is consistent with the weak interaction between *M. tb* PII and AccA3 (Figure 2A). Likewise, the ACC activity of the *M. tb* ACCase 6 complex, which shares the BC-BCCP subunit with ACCase 5, was not affected by the presence of PII either (Figure 2C).

**Figure 3:**
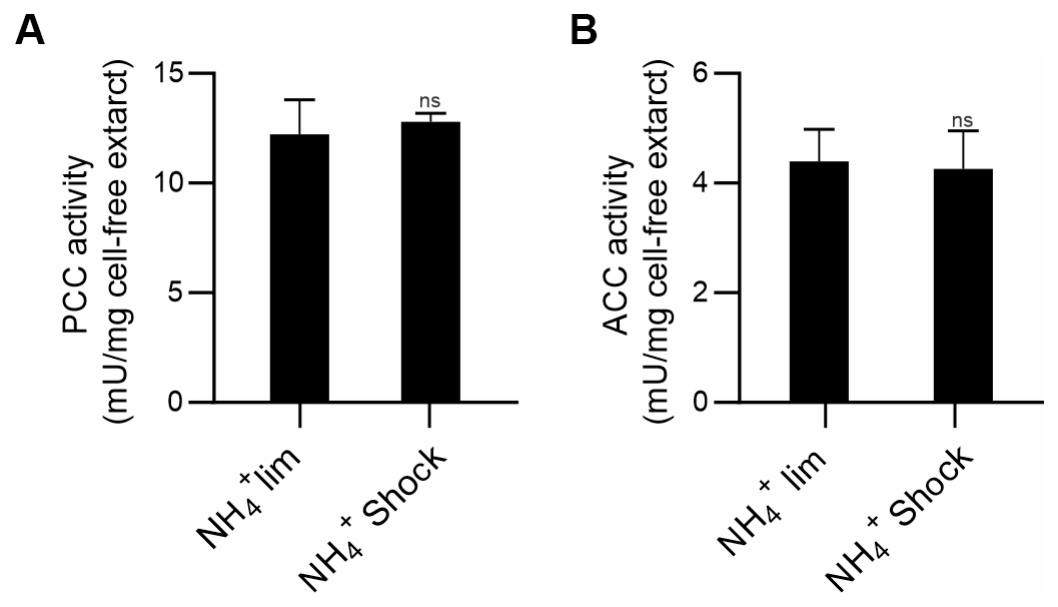
Determination of acyl-CoA carboxylase activities in *M. smegmatis* cell-free extracts. *M. smegmatis* cultures were grown under ammonium-limiting conditions and after ammonium shock, PCC **(A)** and ACC **(B)** activities were determined in cell-free extracts by incorporation of radiolabeled HCO_3_^−^ into the propionyl-CoA or acetyl-CoA as substrates, respectively. The specific activity is plotted as nmols of the corresponding carboxy-acyl-CoA produced per min, per mg of total proteins. Data is reported as the average of quadruplicate and standard deviation. Statistical analysis was performed with Student’s *t*-test at *p* < 0.05, ns: no significant.

In *E. coli* the formation of the ACC-GlnB complex is inhibited when GlnB is uridylated (Gerhardt et al., 2015). Hence, we analyzed ACCase activities in *M. smegmatis* cell-free extracts, grown in limiting nitrogen conditions (1 mM of ammonium) or after the ammonium shock (30 mM of ammonium), conditions were PII is adenylated or de-adenylated, respectively (Figure 1). Under these conditions, no changes were observed in the PCC or the ACC activities of the corresponding ACCases (Figure 3).

### PII is not essential for the viability of *Mycobacterium smegmatis*

**Figure 4:**
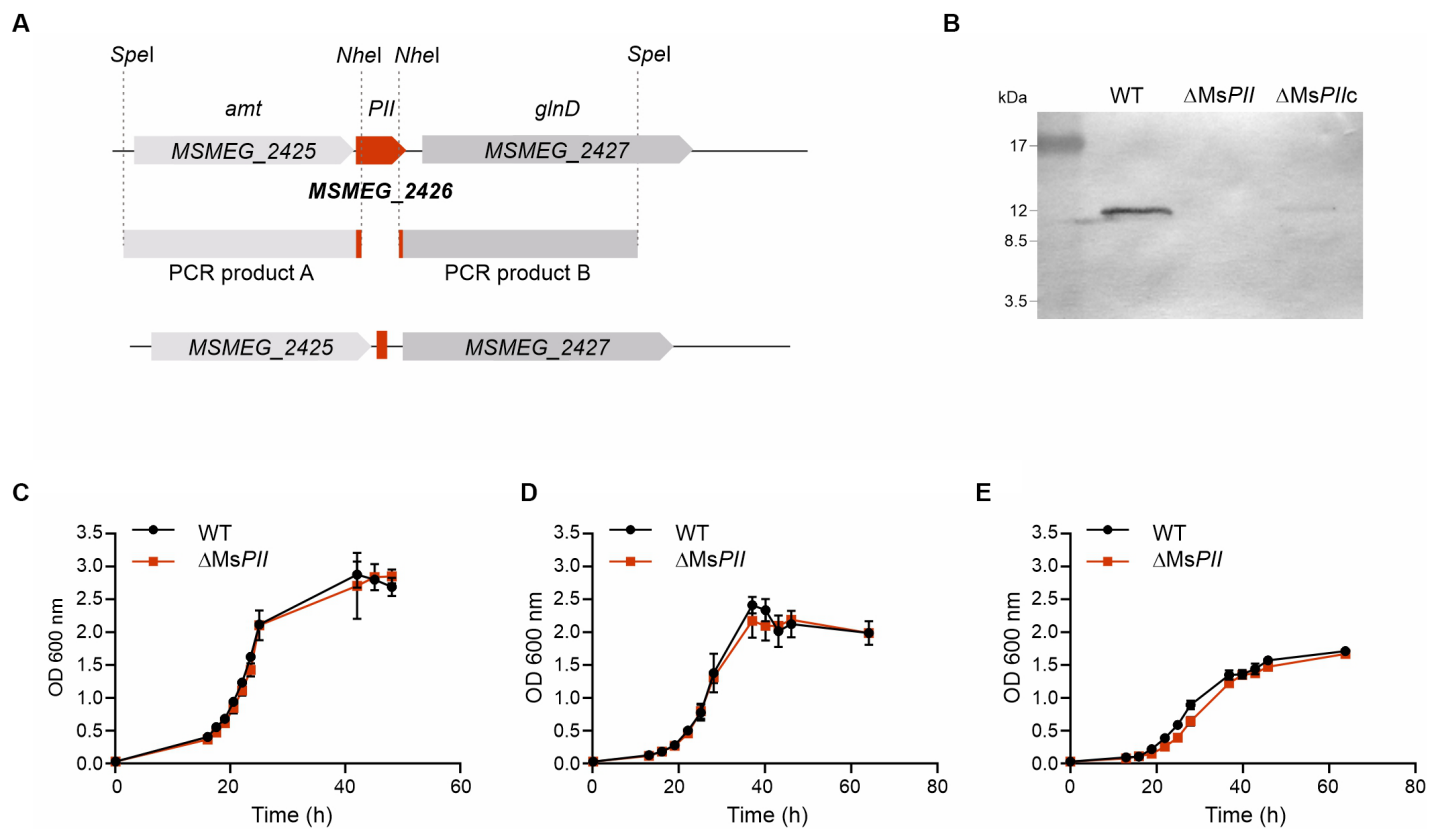
Construction of the *M. smegmatis* PII deletion mutant strain ΔMs*PII*. **(A)** An in-frame deletion of the *PII* gene (*MSMEG_2426*) was constructed by two-steps of homologous recombination with the thermo-sensitive plasmid pDE13. The schematic representation illustrates the genetic *MSMEG_2426* chromosomal region of the wild-type strain mc^2^155, the PCR products A and B, that were amplified and cloned in the delivery vector, and the resulting genetic rearrangement in the mutant strain, obtained for recombination, generating the deletion. **(B)** The presence of the PII protein was analyzed by Western blotting in cell-free extract from the wild type, the ΔMs*PII* and in the ΔMs*PII*c strains. Anti-M. tb antibody was used to detect endogenous PII protein from *M. smegmatis*. Growth curves of *M. smegmatis* mc^2^155 (WT) and ΔMs*PII* strains in rich medium 7H9, complemented with 0.2% glycerol and 0.03% tyloxapol **(C)** or in modified Sauton’s minimal media, supplemented with 15 mM **(D)** or 0.5 mM of (NH_4_)_2_SO_4_ **(E).**

Previous high-throughput studies suggested that PII is not essential for the viability of *M. tb* (Sassetti et al., 2003). Assuming that this would also be applied to *M. smegmatis* PII coding gene (*MSMEG_2426*), we constructed a knockout (KO) mutant in *M. smegmatis* to gain information about the physiological relevance of PII. We disrupted *MSMEG_2426* by using an in-frame deletion strategy, which would not have a polar effect on the expression of *glnD*. For this, we replaced *MSMEG_2426,* the PII coding gene, with a mutant allele, using a classic two-step homologous recombination approach (Crotta Asis et al., 2021; Figure 4A). The strain obtained was named ∆Ms*PII*, confirming that the *MSMEG_2426* gene is not essential for the viability of *M. smegmatis*. PII was not detected by Western blotting in cell-free extracts from two individual colonies of the putative mutants, while PII was perfectly distinguished in cell-free extracts deriving from wild-type strain, which confirmed that the expected allelic exchange at the chromosomal *MSMEG_2426* locus had been successful (Figure 4B). The PII expression levels were partially complemented by the plasmid pDE35, expressing *MSMEG_2426* under the control of the constitutive promoter *P*_hsp60_ (Figure 4B); this strain was named ∆Ms*PIIc*. In all the cases, the anti-*M. tb* PII antibody was used to detect PII proteins from *M. tb* or *M. smegmatis*; this antibody was able to well recognize both proteins (Supplementary Figure S1).

To analyze the physiological consequences of altered PII protein levels, the wild-type, ∆Ms*PII*, strains were grown in 7H9 liquid medium and modified Sauton’s minimal medium supplemented with 15 mM or 0.5 mM of (NH_4_)_2_SO_4_ (Williams et al., 2013, 2015). The growth dynamic of the ∆Ms*PII* strain was very similar to that corresponding to the isogenic wild-type strain (Figures 4C–E). Hence, under these growth conditions, the absence of PII does not modify the cell growth rate.

### The *Mycobacterium smegmatis* ∆Ms*PII* strain has a defective growth in nitrate or nitrite as the sole nitrogen source

**Figure 5:**
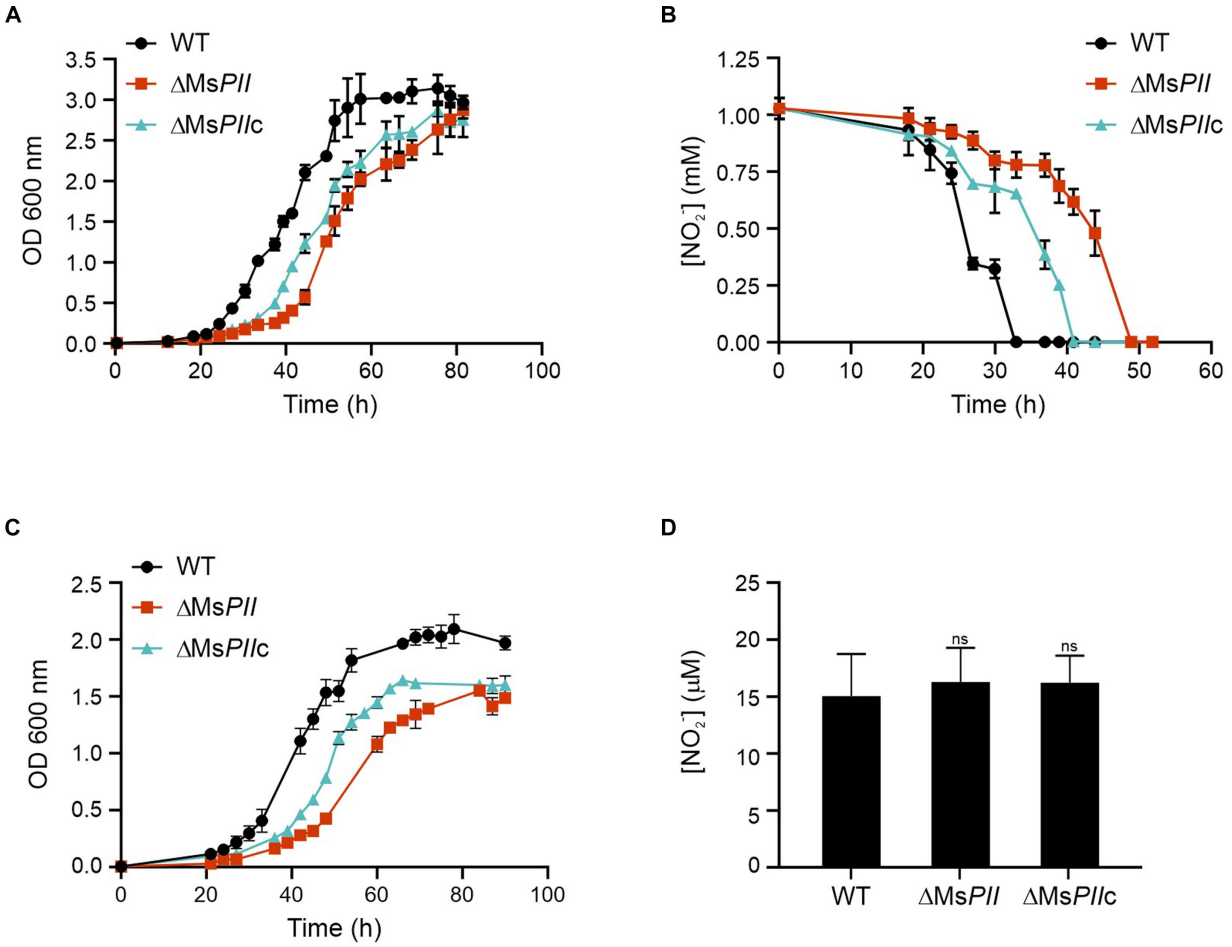
Utilization of nitrite and nitrate as the sole nitrogen source in the *ΔMSPII* strain. High ammonium-cultured cells from wild-type, *ΔMSPII* and *ΔMSPII*c strains were inoculated in fresh modified Sauton’s minimal medium, supplemented with 1 mM of nitrite and growth at 37°C. Bacterial growth **(A)** and nitrite consumption **(B)** were followed. Cell growth was measured using 1 mM nitrate as a nitrogen source **(C)**. The accumulation of nitrite in nitrate-cultures mycobacteria was determined in lysates of the wild-type, PII-null-mutant and *ΔMSPII*c strains **(D)**. Growth was measured as the OD 600 nm, while the nitrite concentration was measured through the Griess assay. Data is expressed as the mean ± S.D. of triplicates. Statistical analysis was performed using Student’s *t*-test at *p* < 0.05, ns, no significant.

It is well described that PII proteins are involved in the regulation of nitrogen metabolism. To further understand the metabolic role of mycobacterial PII, we grew the three strains, wild-type, ∆Ms*PII* and ∆Ms*PIIc* in Sauton’s minimal media where the unique source of nitrogen was nitrate or nitrite. When the medium was supplemented with 1 mM of nitrite (Akhtar et al., 2013) a clear growth delay was observed in the ∆Ms*PII* strain, with a marked and prolonged lag phase, when compared to the wild-type; however, both strains reached similar final ODs. The complemented strain shows an intermediate behavior, suggesting that the expression of the *M. smegmatis* from the plasmid pDE35 partially compensates for the loss of *M. smegmatis* PII expression from the chromosome (Figure 5A). Simultaneously, the concentration of the nitrite remaining in the medium was measured through the Griess assay. The nitrite was not detectable after 33 h in the wild-type cultures, while it took over 41 and 52 h to become undetectable in the ∆Ms*PII*c and ∆Ms*PII* strains, respectively (Figure 5B).

Similar but strongest differences were observed when the strains were grown in Sauton’s media supplemented with 1 mM of nitrate (Figure 5C). The ∆Ms*PII* strain had a reduced growth rate and lower final ODs when compared to the wild type. To determine whether the nitrite assimilation of ∆Ms*PII* cells was the cause for the lower growth rate in nitrate, we measured the nitrite concentration in extracts of cells that were growing in 1 mM nitrate. In this assay, the three cultures resulted in comparable levels of nitrite, indicating the mutant cells are not accumulating or excreting nitrite (Figure 5D).

These experiments suggest that *M. smegmatis* PII protein could be involved in the modulation of nitrate and nitrite assimilation. We conclude that PII is not completely required for cell growth but it increases cell fitness when nitrate or nitrate are used as sole nitrogen sources.

### *Mycobacterium smegmatis* PII deletion mutant presents altered nitrite uptake

**Figure 6:**
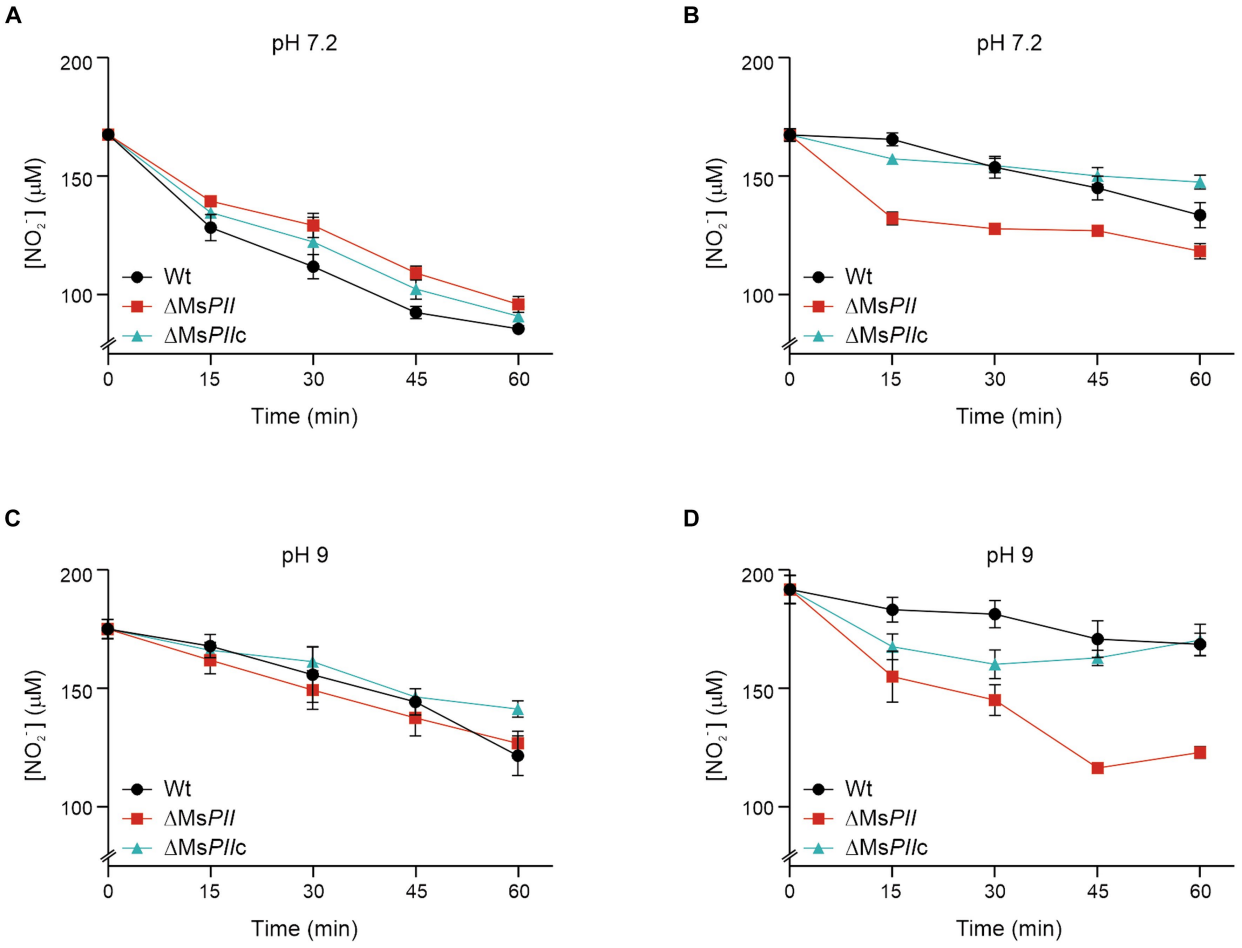
Analysis of nitrite uptake in the wild-type, PII-null mutant and complemented strains. The nitrite uptake was measured in the *M. smegmatis* wild-type, PII-null mutant and complementing strains. The assay was performed on nitrite-grown cells, incubated in the presence of 0.2 mM of NaNO_2_ and in the absence **(A,B)** or presence **(C,D)** of 1 mM of (NH_4_)_2_SO_4_. The data is reported as the average of triplicates ± S.D.

The ability to take up nitrite at different pH was analyzed in wild-type, ΔMs*PII* and ΔMs*PII*c cells, previously adapted to nitrite. The three strains presented a similar uptake activity when nitrite was the sole nitrogen source at pH 7.2 (Figure 6A). The addition of ammonium resulted in a diminution of the nitrite uptake in all the strains; however, this effect was retarded in the ΔMs*PII* strain (Figure 6B). At pH 9, the wild-type strain presented a similar ammonium-dependent inhibition, but this effect was not observed in the ΔMs*PII* strain. The ammonium inhibition resulted slower in ΔMs*PII*c strain (Figures 6C,D). At pH 7.2, nitrite predominantly exists in the form of nitrous acid which could permeate the cell via diffusion. Conversely, at pH 9, the nitrite ions must be transported through the cell envelope by specific permeases (Lee et al., 1998). Therefore, the lack of ammonium-induced inhibition of nitrite uptake at pH 9 in the ΔMs*PII* strain would imply a potential role of *M. smegmatis* PII in orchestrating the hierarchical utilization of ammonium over nitrite as a nitrogen source.

### Activity of nitrate reductase increases at the *Mycobacterium smegmatis* ∆MsPII strain

**Figure 7:**
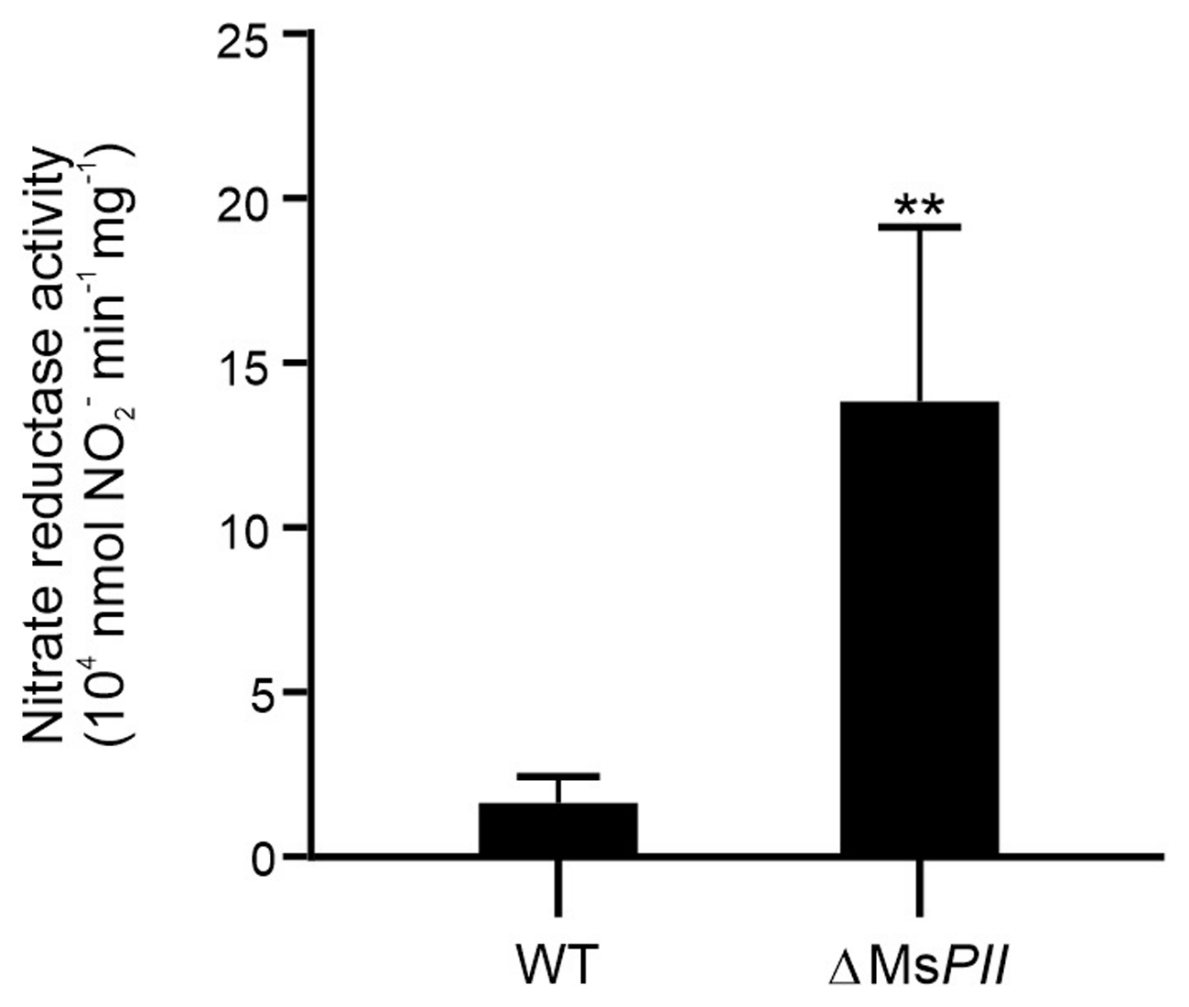
Effect of PII deletion in nitrate reductase activity. Specific nitrate reductase activity was quantified in the cytoplasmic fractions of the cell-free extracts from *M. smegmatis* wild-type and ΔMs*PII* strains. Bacteria were grown aerobically at 37°C in modified Sauton’s minimal medium, containing 1 mM of KNO_3_. Reactions were conducted in presence of the electron donors NADPH and the cofactors FMN and FAD^+^, at pH 7.5 and 30°C. Data are expressed as the mean ± SD of five biological and two technical replicates. Statistical analysis was performed using Student’s *t*-test. ***p < 0.001.

**Figure 8:**
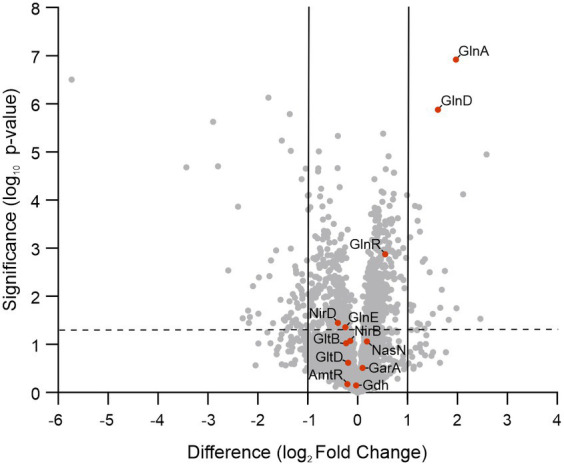
Volcano plot of log_2_ fold changes for ΔMs*PII* versus wild-type strains when grown in 1 mM of nitrate. Orange dots correspond to nitrogen assimilation and metabolism-related proteins. The vertical gray lines represent the log_2_ of the fold change of −2 and 2. The dotted line is the log_10_ of a *p*-value = 0.05.

To assimilate nitrate into the organic nitrogen pool, it undergoes two consecutive reductions. Initially, nitrate is mainly intracellularly reduced to nitrite by the action of a nitrate reductase activity, and subsequently, nitrite is further reduced to ammonia by the reaction catalyzed by the nitrite reductase (Morozkina and Zvyagilskaya, 2007; Gouzy et al., 2014). In mycobacteria, a unique nitrite reductase enzyme was identified (NirBD) (Malm et al., 2009), while several genes encode for proteins with nitrate reductase activity, including NarGHJI, and NarB. A novel type of nitrate reductase was recently described in *M. smegmatis*, which was named NasN. This NADPH-dependent diflavin enzyme is uniquely responsible for nitrate assimilation (Tan et al., 2020; Cardoso et al., 2021). We measured the cytoplasmic nitrate reductase activities in cell–free extracts of *M. smegmatis* wild-type and ∆Ms*PII* strains and found that the protein extracts of the mutant strain had higher nitrate reductase activity than the wild-type (Figure 7). This variation could be the result of protein levels or a direct or indirect regulation of the nitrate reductase activity by PII. NasN is part of the GlnR regulon, the major nitrogen-regulator in *M. smegmatis* (Jeßberger et al., 2013). Even though there is no evidence that PII and GlnR may interact, PII could be modulating protein expression as seen in other systems (Huergo et al., 2013). Therefore, we compared the protein profiles of both strains when grown in 1 mM nitrate by LC–MS/MS. The proteins with significant differences were screened according to the criteria of │FC│ > 1.5 and *p* < 0.05 (Figure 8). Though, the amount of a few proteins involved in nitrogen metabolism was altered (Table 2), no differences were observed in the GlnR or NasN levels between these strains, suggesting that the modulation of nitrate reductase activity by PII occurs by posttranslational mechanism. The subunits of the NirBD complex, which is under the regulation of GlnR and NasR, were equally represented in both samples as well. The comprehensive roster of differentially expressed proteins is detailed in Supplementary Tables S1, S2.

**Table 2:** Proteins involved in nitrogen metabolism exhibit differential expression during growth in 1 mM KNO_3_ in the *M. smegmatis* ΔMs*PII* strain in comparison with the wild-type.

**Locus**	**Gene name**	**Fold Change**	***p*-value**	**Description**
MSMEG_5119	*pruA*	6.001362186	1.13206E-05	L-glutamate gamma-semialdehyde dehydrogenase
MSMEG_0019		3.950896355	0.01779877	Phenyloxazoline synthase MbtB
MSMEG_2427	*glnD*	3.059368826	1.33423E-06	Bifunctional uridylyltransferase/uridylyl-removing enzyme
MSMEG_6591		2.847334688	0.023175276	Aminotransferase, class V family protein
MSMEG_6266		2.374394323	0.000453635	Thiocyanate hydrolase beta subunit
MSMEG_6267		2.353291727	0.000140793	Thiocyanate hydrolase gamma subunit
MSMEG_2493		2.219032007	0.00013309	Aminotransferase, class I and II family protein
MSMEG_1295		2.075248595	0.01497605	5-hydroxyisourate hydrolase
MSMEG_1612		−2.132949783	0.004773754	Extracellular solute-binding protein, family protein 3
MSMEG_0965; MSMEG_5483; MSMEG_0520; MSMEG_6057	*mspA, mspC; mspB; mspD*	−2.356115069	0.030465619	Porin MspA; Porin MspC; Porin MspB; Porin MspD
MSMEG_0435		−2.375975566	0.002398612	Allophanate hydrolase subunit 2
MSMEG_0435		−2.375975566	0.002398612	Allophanate hydrolase subunit 2
MSMEG_3056		−2.455250117	0.009071936	ABC transporter ATP-binding protein
MSMEG_5368	*ehuB*	−2.656561294	0.026949456	Ectoine/hydroxyectoine ABC transporter solute-binding protein
MSMEG_5371	*ehuA*	−3.238464659	0.036432586	Ectoine/hydroxyectoine ABC transporter, ATP-binding protein
MSMEG_3973		−3.444703955	7.43396E-07	*N*-methylhydantoinase
MSMEG_1416		−53.04335044	3.14673E-07	Pyridine nucleotide-disulfide oxidoreductase
MSMEG_2426^a^	*PII*	−295.1715778	8.16094E-08	Nitrogen regulatory protein PII
MSMEG_2128^a^	*mbtK*			Lysine *N*-acyltransferase
MSMEG_3787^a^				D-aminoacylase

^a^Protein only detected in wild-type strain.

The enrichment analysis using a Fisher’s exact test^1^ (Ge et al., 2018) of the significantly changed proteins screened from the LC–MS/MS showed that proteins involved in ammonium assimilation pathways were increased, including GlnA and GlnD, as well as proline and arginine metabolism (Supplementary Figure S4). On the other hand, many transmembrane transporters, including putative amino acid ABC transporters, were reduced. Surprisingly, one of these was the Mce4 transport system involved in cholesterol uptake (García-Fernández et al., 2017; Rank et al., 2021), and which is considered a virulence factor in *M. tb*. In addition, the expression of proteins related to the stress response was also altered.

### Mycobacterial PII interacts with the ammonia transporter Amt

In many bacteria and Archaea, the PII protein named GlnK, interacts with the ammonia channel AmtB to modulate its function (Thomas et al., 2000; Coutts and Thomas, 2002; Sant'Anna et al., 2009). When the cellular nitrogen status increases, the GlnK-AmtB binding promotes a block of ammonia transport into the cell (Coutts and Thomas, 2002; Zheng et al., 2004; Conroy et al., 2007). Regulation of AmtB activity seems to be the archetypical function of the GlnK protein, also these genes form a conserved *glnK-amtB* operon in a range of prokaryotes.

Based on both synteny and protein crystal structure analyses, it has been previously proposed that *M. tb* GlnB may in fact share an analogous function to *E. coli* GlnK and not GlnB (Shetty et al., 2010). However, the precise function of PII in mycobacteria has not been studied at a biochemical level.

**Figure 9:**
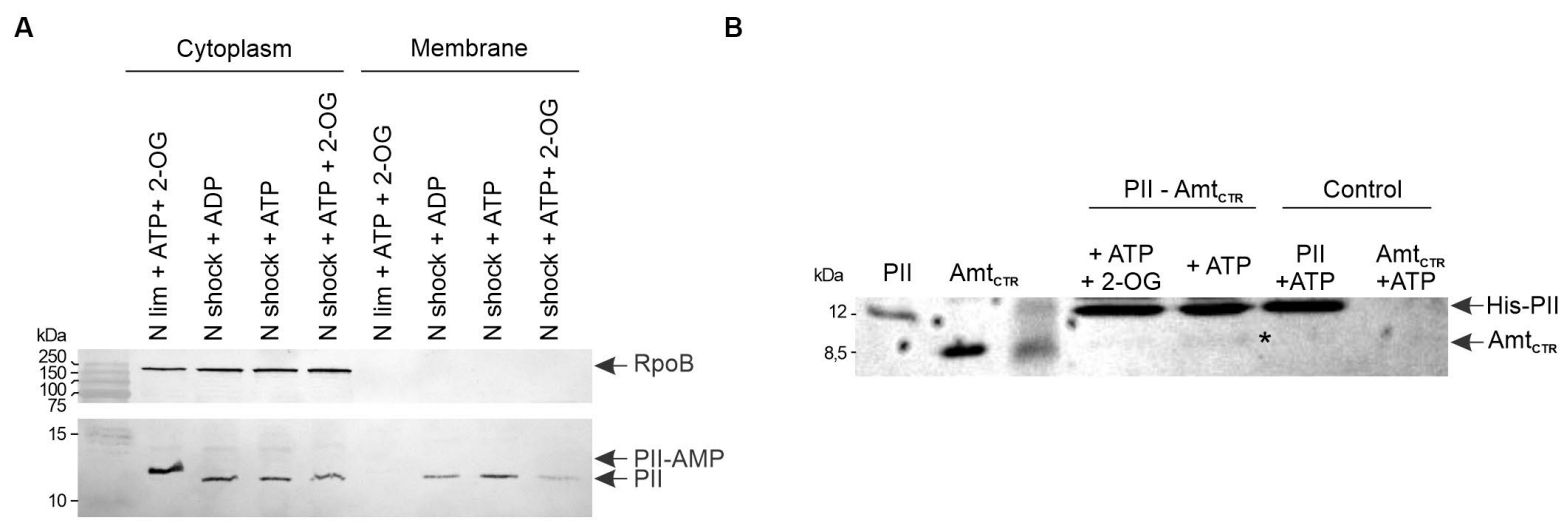
Interaction between PII and Amt, and effect of ammonium concentration on PII cellular localization. **(A)** The cellular localization profiles of PII in cell-free extracts from wild-type *M. smegmatis* were analyzed after an ammonium shock (NH_4_^+^), in the presence of the PII allosteric effectors, as indicated: 4.5 mM ATP, 0.6 mM ADP and/or 1.5 mM 2-OG. Cytoplasmic and membrane fractions were subjected to SDS-PAGE followed by Western blotting with anti-*M. tb* PII and RpoB antibodies**. (B)** The interaction of *M. tb* PII with the C-terminal cytosolic region of Amt (Amt_CTR_) was analyzed by a pull-down assay. Purified His-PII was mixed with purified Amt from *M. tb.* Proteins eluted from the Ni^2+^ resin were analyzed by SDS-PAGE and the gel was stained with Coomassie Blue.

To verify if *M. tb* PII function as GlnK interacting with Amt, we analyzed the presence of PII on the membrane and cytoplasmic fractions by SDS-PAGE and Western blotting. Cells were grown in ammonium–limiting conditions for 28 h. An aliquot of the culture was incubated in 30 mM of ammonium for 15 min. The membranes were isolated by centrifugation and washed in the presence of ATP, ADP, or ATP and 2-OG. PII was only present in the cytoplasmic fraction in the ammonium-limiting condition. In contrast, after the ammonium shock, PII was found in both the cytoplasmic and membrane fractions, indicating that PII associates with the membrane in an ammonium-dependent manner. This association was weakened by the presence of the PII protein allosteric effectors ATP and 2-OG (Figure 9A).

To determine if the association with the membrane was through Amt transporter, we performed a pull-down assay in which purified *M. tb* His_6_-PII was mixed with a Ni-NTA resin (Novagen) and incubated with purified C-terminal Amt (Amt_CTR_), without tags, for 30 min. After three washes with buffer containing different combination of the PII protein allosteric effectors Mg-ATP and 2-OG, the elutions were analyzed by SDS-PAGE. Figure 9B shows that PII is able to interact with Amt_CTR_ being negatively modulated by 2-OG, in agreement with the PII cellular localization analysis (Figure 9A). These data confirm that *M. tb* GlnB plays an ancestral role as a GlnK protein, in addition to its function as a modulator of nitrite assimilation.

## Discussion

Structural and biochemical investigations have been conducted on the PII proteins of mycobacteria *M. tb* and *M. smegmatis*; however, their specific functions remain elusive. A critical aspect in unraveling this role consists in elucidating their capacity to perceive nitrogen, carbon, and energy status. Like in other actinomycetes, it has been demonstrated that mycobacterial PII is adenylated upon induction by ammonium starvation (Williams et al., 2013). We verified that this modification is rapidly reversible in response to changes in ammonium levels in *M. smegmatis* (Figure 1). Moreover, we observed that PII can be localized at the membrane following an ammonium shock; with the PII membrane interaction being regulated by 2-OG (Figure 9A). This result is consistent with the PII: AmtB interaction that modulates the incorporation of ammonium through the AmtB channel. When ammonium is scarce, GlnK binds the allosteric effectors ATP and 2-OG and GlnK remains located in the cytosol allowing full AmtB activity. Conversely, when ammonium becomes available, GlnK binds the allosteric effector ADP and migrates to the cell membrane to obstruct the AmtB transporter via the interaction with the GlnK T-loop (Coutts and Thomas, 2002; Zheng et al., 2004; Conroy et al., 2007). The hypothesis the mycobacterial PII regulates Amt activity is reinforced by the genomic context of the PII gene (*amt-PII-glnD*), the conservation of amino acid residues involved in the interaction between GlnK and AmtB in *E. coli,* in both *M. tb* and *M. smegmatis* (Conroy et al., 2007) and the resolution of *M. tb* PII crystal structure (Shetty et al., 2010). Also, a homology model based on the crystal structure of the *E. coli* GlnK: AmtB complex showed that *M. tb* PII T-loop could engage in complex formation with AmtB (Shetty et al., 2010). Even though we were unable to heterologously express *M. tb* Amt in the soluble fraction of *E. coli*, we could observe an interaction between *M. tb* PII and the cytosolic C-terminal domain of Amt (Figure 9B). Previous studies have also reported that Ec-GlnK shows a reduced interaction with the C-terminal truncated version of AmtB (Coutts and Thomas, 2002). Taken together, these data provide strong evidence that the mycobacteria PII protein retained the archetypical GlnK-like function being able to regulate Amt activity by physical interaction with the Amt C-terminal domain, upon an ammonium shock.

Acetyl-CoA carboxylase complexes are also targets of PII regulation in many organisms (Feria Bourrellier et al., 2010; Gerhardt et al., 2015; Hauf et al., 2016; Rodrigues et al., 2019; Wang et al., 2021). We found that *M. tb* PII weakly interacts with the ACCase subunit AccA3, which contains the BCCP domain. However, this weak interaction did not affect ACC or PCC activities of the main complexes ACCases 5 and 6. As shown in Figure 2A, AccA3 from *M. tb* can bind GlnB protein from other species. This interaction seems stronger with GlnB from *A. brasiliensis* and it proved to inhibit its PCC activity (Supplementary Figure S3). In *E. coli,* both GlnB and GlnK, can interact with the BCCP subunit of the ACC complex, however, only GlnB regulates ACC activity *in vitro* (Gerhardt et al., 2015). These data support that the GlnB-like PII evolved to regulate fatty acid biosynthesis in agreement with a recent report from *Streptomyces hygroscopicus* var. *ascomyceticus* (Wang et al., 2021). This particular actinomycete encodes two PII proteins, named Sh-GlnK and Sh-GlnB. However, only Sh-GlnB has the ability to modulate ACCase activity in this microorganism. The fact that mycobacteria PII was not able to regulate ACC/PCC activities also suggests that it evolved to act as a GlnK-type of PII.

To gain deeper insights into the role of PII in mycobacteria, we decided to construct KO mutants. A high-throughput analysis carried out by transposon site hybridization (TraSH) indicated that *M. tb glnB* gene is not essential (Sassetti et al., 2003); however, after several attempts, we were unable to construct a deletion mutant in H37Rv strain. Similarly, Read et al. (2007) also failed to obtain a *glnB* mutant strain. However, we successfully obtained a PII mutant strain in *M. smegmatis* (ΔMs*PII*) by double homologous recombination, which presented normal growth in a media supplemented with low concentrations of ammonium. This result suggests that PII must not play an essential role in adaptation to low ammonium levels. In addition, the ΔMs*PII* strain did not present any growth difference in media with increased levels of nitrogen, when compared with the wild-type strain, indicating that the regulation of the Amt channel is not required for bacteria survival (Supplementary Figure S5).

The ΔMs*PII* mutant strain was also exposed to various organic and inorganic nitrogen sources, exhibiting a slight reduction of optical densities when glutamate or asparagine were used as sole nitrogen sources (Supplementary Figure S6). The most prominent defect of the ΔMs*PII* strain was the impairment to grow when nitrate or nitrite were used as the sole nitrogen source, primarily characterized by extended lag phases (Figure 5). Unlike the wild-type strain, nitrite uptake was not inhibited by ammonium in the ΔMs*PII* strain (Figure 6). This strain also presented higher nitrate reductase activity (Figure 7) and increased levels of glutamine synthetase when nitrate was the sole nitrogen source. These behaviors resemble those reported in cyanobacteria PII mutant strains (Forchhammer and De Marsac, 1995; Lee et al., 1998; Kobayashi et al., 2005). In photosynthetic bacteria, ammonium is the preferred nitrogen source and it negatively regulates the assimilation of alternative nitrogen sources, such as nitrate or nitrite, via the PII protein. A PII-null mutant strain of *Synechococcus* sp. PCC 7942 exhibited a slight decrease in growth rate when exposed to nitrate or glutamine. This was accompanied by an increase in nitrate reductase activity compared to the wild-type, along with elevated levels of *glnA* expression at both transcript and protein levels (Forchhammer and De Marsac, 1995). Glutamine synthetase accumulation was also observed in an *E. coli* mutant lacking GlnB (Bueno et al., 1985), regardless of the nitrogen status. In both, *E. coli* and cyanobacteria glutamine synthetase and *E. coli’s glnD* expression depends on transcriptional regulators, whose activity is indirectly modulated by GlnB (Jiang and Ninfa, 1999; Espinosa et al., 2006, 2007). In the case of *M. smegmatis, glnD* and *glnA* are under GlnR control (Jenkins et al., 2013; Jeßberger et al., 2013), but little is known about how GlnR is activated. Overexpression of GlnD and GlnA in the ΔMs*PII* strain could be the result of an attempt to compensate for the absence of PII. However, in the ΔMs*PII* mutant, only a subset of genes corresponding to the GlnR operon exhibited altered protein levels. In addition, in the *M. tuberculosis’ glnD*-null mutant, the expression of the *amt-glnB-glnD* operon is upregulated under nitrogen deficiency conditions (Read et al., 2007). Therefore, the possibility that PII is involved in the transcription regulation of these genes, through its interaction with as-yet-undetermined targets, cannot be ruled out.

In contrast to the *M. smegmatis* ΔMs*PII*, for *Synechococcus* sp. PCC 7942, nitrate-cultured mutant cells exhibited an exacerbated nitrite excretion. The PII-null mutant also displayed normal nitrite uptake activity at pH 7.2, but high assimilation of nitrate even in the presence of ammonium at high pH (9.6), bypassing the ammonium repression. Consequently, the modulation of the nitrate/nitrite uptake in *M. smegmatis* could potentially be attributed to an effect of PII on ABC transporters, as was observed for other systems (Watzer et al., 2019). However, the fact that the ammonium-inhibition takes longer to occur in the mutant strain at pH 7.2 suggests additional regulatory points in the process of nitrite assimilation that may also be under the influence of PII.

In *M. smegmatis*, NarK and NarK3 were designed to be involved in nitrate and nitrite transport (Amon et al., 2009). Nevertheless, there are numerous putative subunits for ABC transporters that are indicated to be involved in nitrate/nitrite assimilation. Further research should be carried out to thoroughly understand this mechanism. It may be interesting to analyze whether the role of PII in nitrate/nitrite assimilation is replicated in *M. tuberculosis,* considering that nitrate has a relevant role in virulence (Gouzy et al., 2013). Also, the PII-null mutant strain resulted defective in replication during the invasion of mouse macrophages (Rengarajan et al., 2005).

**Figure 10:**
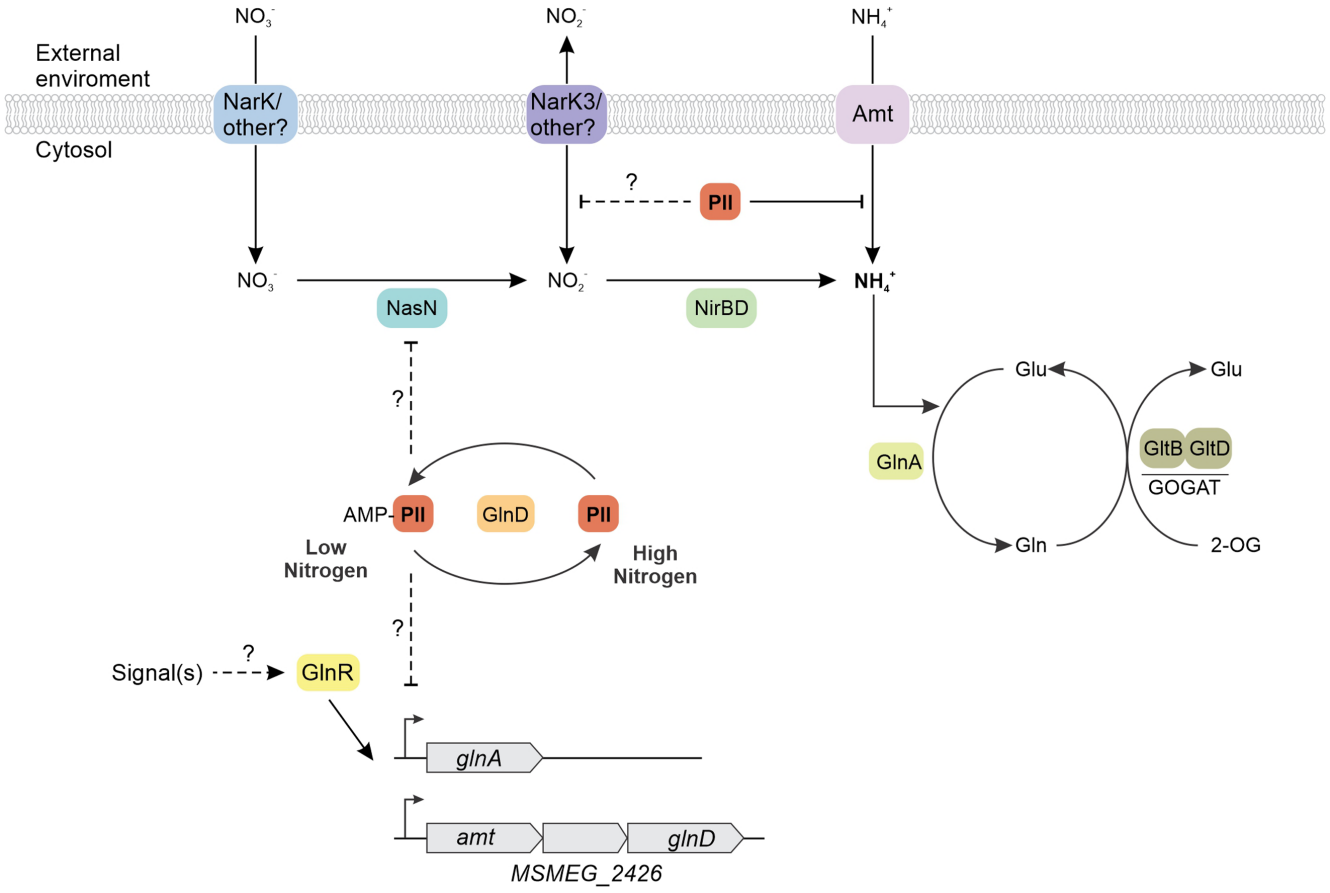
Proposed model about physiological roles of PII protein in mycobacteria. The putative ammonium transporter Amt mediates the ammonium (NH_4_^+^) enter to the bacterial cytosol in a low nitrogen regimen. The glutamine synthetase GlnA assimilates the ammonium to synthesize glutamine, which serves as the substrate for the glutamine oxoglutarate aminotransferase (GOGAT) in the synthesis of glutamate. The bacteria sense nitrogen status using the 2-OG/glutamine ratio. When ammonium is scarce, PII binds the allosteric effectors ATP and 2-OG and it is adenylated. PII-AMP remains located in the cytosol allowing full Amt activity. Conversely, when ammonium becomes available, PII is deadenylated and binds the allosteric effector ADP, migrating to the cell membrane and obstructing the Amt transporter via the interaction with the PII T-loop. The absence of PII, like in the ΔMs*PII* mutant strain, produces a growth defect when nitrate or nitrite were used as the sole nitrogen source. The ΔMs*PII* mutant strain presented higher nitrate reductase activity and increased levels of glutamine synthetase when nitrate was the sole nitrogen source.

Altogether, the PII proteins of mycobacteria maintain their role as modulators of ammonium entry, although this function does not seem to be essential for the organism. However, their absence showed negative effects when cells are grown in nitrate or nitrite, indicating that PII is involved in the uptake or metabolization of these alternative nitrogen sources, as we indicate in a proposed model (Figure 10). It is worth noting the flexibility of PII proteins to regulate different groups of proteins depending on the specie, making in some cases the distinction between GlnK and GlnB types to be blurry.

## Data availability statement

The mass spectrometry proteomics data have been deposited to the ProteomeXchange Consortium via the PRIDE (Perez-Riverol et al., 2022) partner repository with the dataset identifier PXD049128 and 10.6019/PXD049128.

## Author contributions

DE: Data curation, Formal analysis, Investigation, Methodology, Software, Visualization, Writing – original draft. EG: Formal analysis, Writing – review & editing, Investigation, Methodology. LR: Investigation, Formal analysis, Writing – review & editing, Methodology. LH: Conceptualization, Data curation, Formal analysis, Supervision, Writing – review & editing. HG: Conceptualization, Data curation, Formal analysis, Supervision, Writing – review & editing, Funding acquisition. LD: Conceptualization, Data curation, Formal analysis, Funding acquisition, Supervision, Writing – review & editing, Project administration, Resources, Validation, Visualization, Writing – original draft.

## Footnotes

^1^ShinyGO 0.77: http://bioinformatics.sdstate.edu/go/
