# Supplementary material for: The PII protein interacts with the Amt ammonium transport and modulates nitrate/nitrite assimilation in mycobacteria
Source: Front Microbiol. 2024 Mar 25;15:1366111. doi: 10.3389/fmicb.2024.1366111 (PMC11001197; doi:10.3389/fmicb.2024.1366111)
Supplement: Supplementary file 1 [file Data_Sheet_1.pdf]

The PII protein interacts with the Amt ammonium transport and modulates nitrate/nitrite assimilation in mycobacteria

Supplementary material

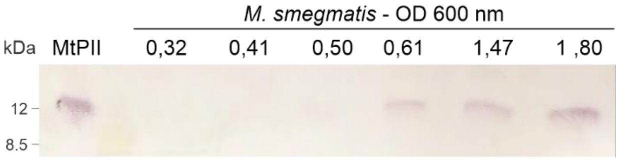

Supplementary Figure 1.
M. tub purified PII (Mt-PII) and endogenous *M. smegmatis* PII in cell-free extracts, grown in 0.5 mM $(NH_4)_2SO_4$, were detected by Western blotting using an anti-*M. tub* PII antibody.

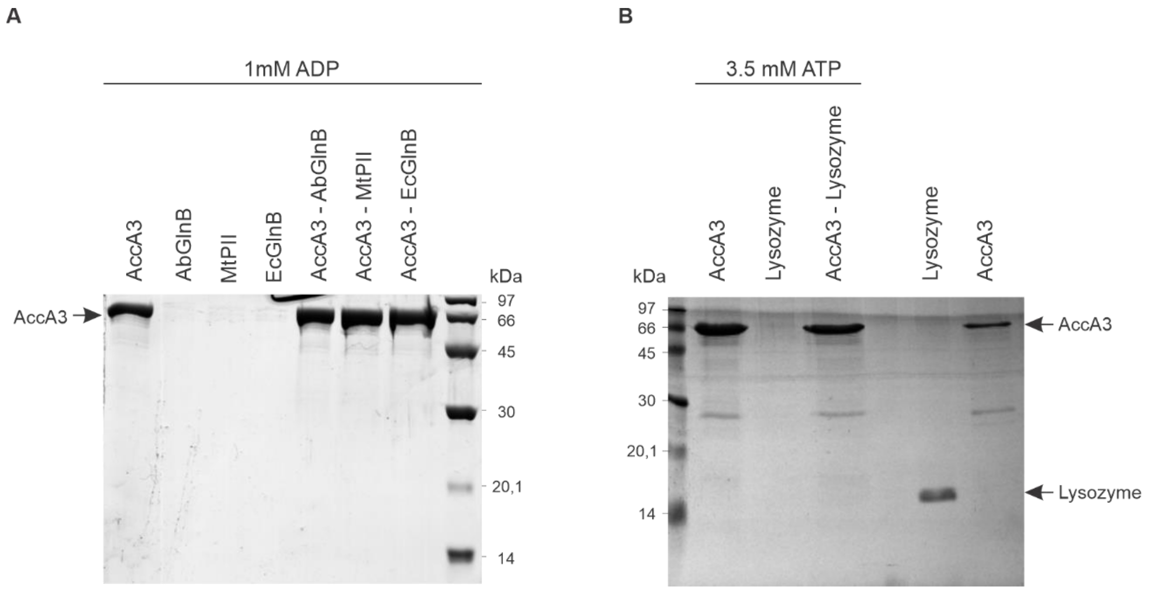

Supplementary Figure 2.

Specificity of the interaction between AccA3 and PII. The complex formation between *M. tb* His-AccA3 and *M. tb* PII in the presence of 1 mM ADP **(A)** or lysozyme under saturating ATP concentrations (3.5 mM) **(B)** was assessed by pull-down and analyzed by SDS-PAGE. In **B**, the last two lines are controls of migration of purified proteins, Lysozyme and AccA3, respectively.

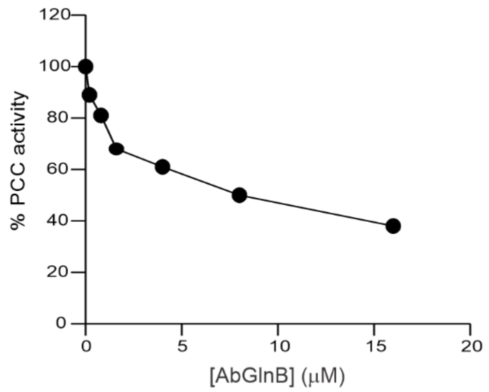

Supplementary Figure 3.

PCC activity of ACCase 5 complex in the presence of variable concentrations of GlnB form *A. brasilense* (AbGlnB). The activity is plotted as a percentage considering 100% activity when AbGlnB wss not included.

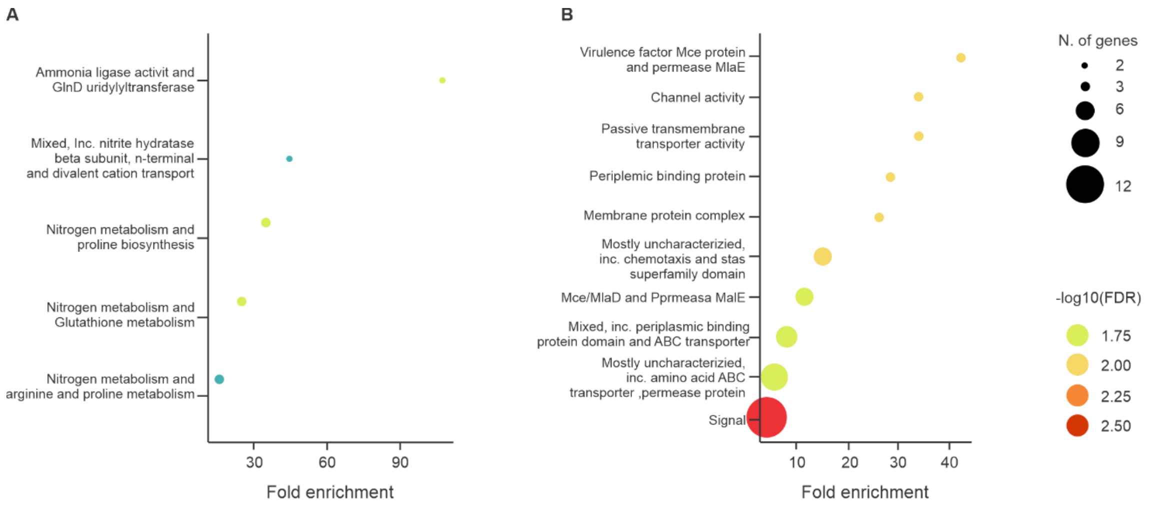

Supplementary Figure 4.

Enrichment analysis of proteins that were differentially upregulated **(A)** and downregulated **(B)** in the Δ*MsPII* mutant strain compared to the wild type. The data were analyzed using Fisher's test with a false discovery rate (FDR) of 0.05.

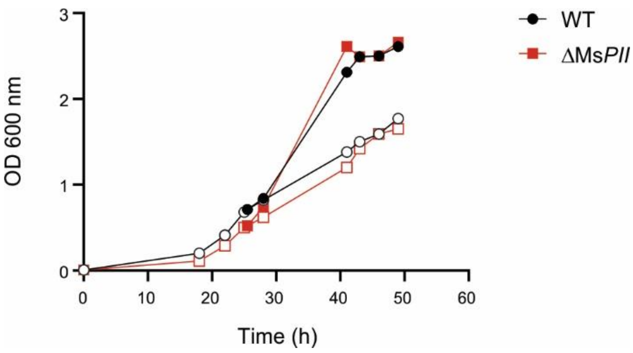

Supplementary Figure 5.

Growth of *M. smegmatis* wild-type and ΔMSPII after an ammonium shock. Cultures were grown in modified Sauton´s minimal medium supplemented with 0.5 mM $(NH_4)_2SO_4$ (open symbols) and at 25 h, a concentration of 15 mM $(NH_4)_2SO_4$ was added to induce an ammonium shock (closed symbols).

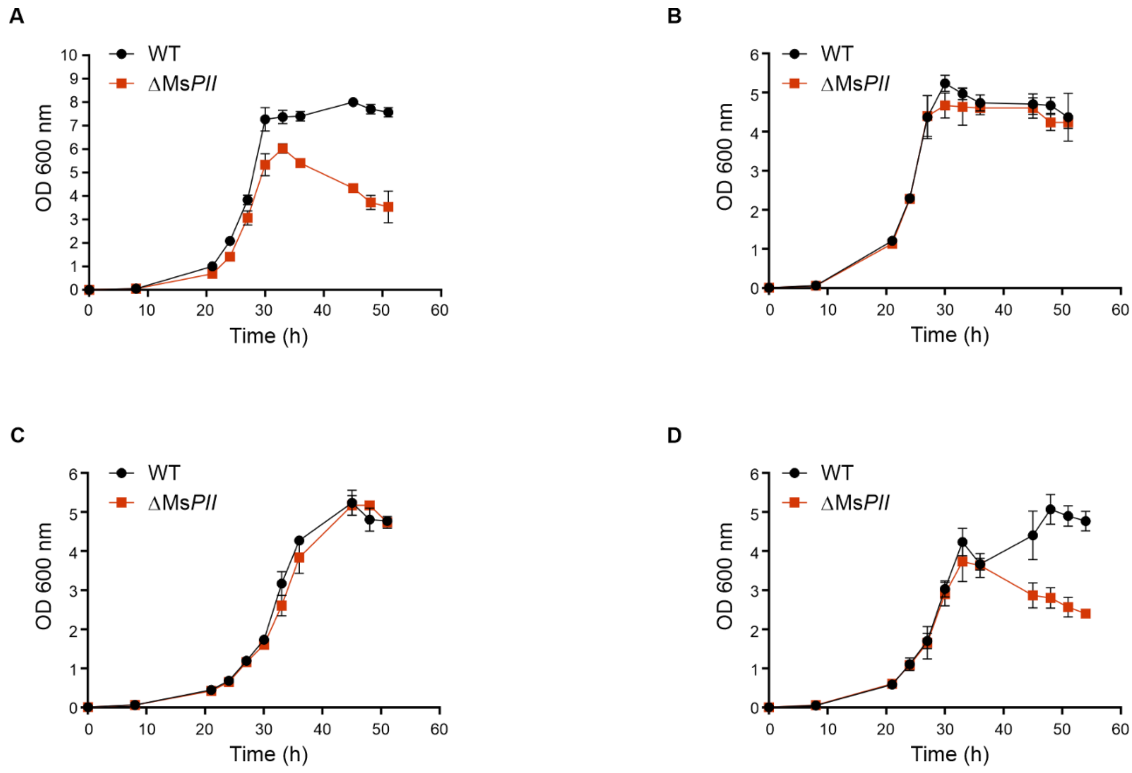

Supplementary Figure 6.
Growth of *M. smegmatis* PII-null mutant in different aminoacids as unique sources of nitrogen. Ammonium-grown cell from the wild-type and ΔMsPII strain were grown in modified Sauton´s minimal medium supplemented with 34 mM of glutamate **(A)**, glutamine **(B)**, aspartate **(C),** or asparagine **(D)** as the sole nitrogen source. The data is reported as the average of triplicates ± S.D.

Supplementary Table 1.

Proteins that are differentially increased during growth in 1 mM KNO_3 in the *M. smegmatis* ΔMs*PII* strain in comparison with the wild-type strain.

Locus	Gene Name	Fold Change	*p*-value	Description
MSMEG_5119	*pruA*	6.0013	0.00001	L-glutamate gamma-semialdehyde dehydrogenase
MSMEG_2417		5.5156	0.02930	DUF177 domain-containing protein
MSMEG_1351		4.3317	0.00008	Cyclopropane-fatty-acyl-phospholipid synthase
MSMEG_0019		3.9509	0.01780	Phenyloxazoline synthase MbtB
MSMEG_4290	*glnA*	3.9215	0.00000	Glutamine synthetase
MSMEG_5155		3.5490	0.03075	Nitroreductase
MSMEG_1749		3.3602	0.00299	Putative monooxygenase
MSMEG_5557		3.2232	0.00734	Secreted protein
MSMEG_0383	*rv1174c*	3.1543	0.01724	Rv1174c
MSMEG_2427	*glnD*	3.0594	0.00000	Bifunctional uridylyltransferase/uridylyl-removing enzyme
MSMEG_6591		2.8473	0.02318	Aminotransferase, class V family protein
MSMEG_0230		2.7251	0.00319	CopY family transcriptional regulator
MSMEG_3449		2.6697	0.03704	DNA-binding protein
*MSMEG_3435**		2.5493	0.00170	ParD-like antitoxin of type II toxin-antitoxin system
MSMEG_6343		2.5352	0.04707	Phosphoesterase
MSMEG_6361		2.4763	0.00191	Transcriptional regulator, MarR family protein
MSMEG_1961		2.3810	0.03868	Tat pathway signal protein
MSMEG_6266		2.3744	0.00045	Thiocyanate hydrolase beta subunit
MSMEG_6267		2.3533	0.00014	Thiocyanate hydrolase gamma subunit
MSMEG_4176		2.3050	0.03641	ATPase
MSMEG_0898		2.3020	0.00027	Tagatose-1,6-bisphosphate aldolase GatY
MSMEG_2493		2.2190	0.00013	Aminotransferase, class I and II family protein
MSMEG_5231		2.1664	0.01579	UPF0113 domain-containing protein
MSMEG_6354		2.1052	0.01994	Serine esterase, cutinase family protein
MSMEG_1950		2.0762	0.00059	General stress protein CsbD
MSMEG_1295		2.0752	0.01498	5-hydroxyisourate hydrolase

Supplementary Table 2.

Proteins that are differentially decreased during growth in 1 mM KNO_3 in the *M. smegmatis* ΔMs*PII* strain in comparison with the wild-type strain.

Locus	Gene Name	Fold Change	*p*-value	Description
MSMEG_2183		-2.0165	0.0018	NTP_transf_2 domain-containing protein
MSMEG_5898	*mce4C*	-2.0333	0.0402	Virulence factor Mce family protein
MSMEG_0418	*sdhA*	-2.0565	0.0000	Succinate dehydrogenase flavoprotein subunit
MSMEG_2110		-2.0616	0.0254	DUF2993 domain-containing protein
MSMEG_1655		-2.1191	0.0154	Hydrolase, alpha/beta fold family protein, putative
MSMEG_1612		-2.1329	0.0048	Extracellular solute-binding protein, family protein 3
MSMEG_2466		-2.1464	0.0097	Glutaryl-CoA dehydrogenase
MSMEG_3266	*eltP*	-2.1515	0.0113	Erythritol/L-threitol-binding protein
MSMEG_2112		-2.1559	0.0091	Secreted protein
MSMEG_1547		-2.1694	0.0035	Glycerol dehydratase large subunit
MSMEG_5890		-2.1748	0.0000	Transcriptional regulator
MSMEG_1883	*opuD*	-2.1943	0.0419	Glycine betaine transporter
MSMEG_2079		-2.2001	0.0070	Alcohol dehydrogenase
MSMEG_3270		-2.2532	0.0165	SN-glycerol-3-phosphate ABC transporter, ATP-binding protein
MSMEG_5896	*mce4E*	-2.2723	0.0379	Virulence factor Mce family protein
MSMEG_2381		-2.2809	0.0061	Peptidase_S9 domain-containing protein
MSMEG_4940	*atpF*	-2.2843	0.0174	ATP synthase subunit delta
MSMEG_5897	*mce4D*	-2.3202	0.0139	Virulence factor Mce family protein
MSMEG_1374		-2.3243	0.0408	Ribose ABC transporter, periplasmic binding protein
MSMEG_3419		-2.3474	0.0034	Ferritin-like domain-containing protein
MSMEG_2634		-2.3507	0.0029	NMT1 domain-containing protein
MSMEG_0965; MSMEG_5483; MSMEG_0520; MSMEG_6057	*mspA, mspC; mspB; mspD*	-2.3561	0.0305	Porin MspA; Porin MspC; Porin MspB; Porin MspD
MSMEG_0435		-2.3760	0.0024	Allophanate hydrolase subunit 2
MSMEG_2014	*modC*	-2.3904	0.0108	Molybdenum import ATP-binding protein
MSMEG_3056		-2.4553	0.0091	ABC transporter ATP-binding protein

Supplementary Table 2. Continued

Locus	Gene Name	Fold Change	p-value	Description
MSMEG_2007		-2.4602	0.0328	Putative HpcE protein
MSMEG_4939	*atpFH*	-2.4754	0.0355	synthase subunit b-delta
MSMEG_4656		-2.5154	0.0223	Sugar ABC transporter ATP-binding protein
MSMEG_6212		-2.5253	0.0010	Hemerythrin HHE cation binding domain subfamily protein, putative
MSMEG_2346		-2.5273	0.0000	Phytoene synthase
MSMEG_4256		-2.5504	0.0436	NLP/P60 family protein
MSMEG_1713	*araB*	-2.5637	0.0000	Ribulokinase
MSMEG_5228		-2.5765	0.0128	3-beta hydroxysteroid dehydrogenase/isomerase family protein
MSMEG_4561		-2.5986	0.0273	ABC Fe3+-siderophores transporter, periplasmic binding protein
MSMEG_6319		-2.6066	0.0444	Penicillin-binding protein, transpeptidase
MSMEG_5368	*ehuB*	-2.6566	0.0269	Ectoine/hydroxyectoine ABC transporter solute-binding protein
MSMEG_3855		-2.6870	0.0271	Lipoprotein
MSMEG_0976		-2.7391	0.0319	CcoQ/FixQ family Cbb3-type cytochrome c oxidase assembly chaperone
MSMEG_1546		-2.7835	0.0302	Coenzyme B12-dependent glycerol dehydrogenase small subunit
MSMEG_1773		-2.8663	0.0000	Iron-containing redox enzyme family protein
MSMEG_1704		-2.9152	0.0119	ABC transporter
MSMEG_1758		-3.0988	0.0011	Ribonuclease
MSMEG_5371	*ehuA*	-3.2385	0.0364	Ectoine/hydroxyectoine ABC transporter, ATP-binding protein
MSMEG_3302		-3.3294	0.0018	Short-chain dehydrogenase/reductase
MSMEG_2524		-3.4034	0.0038	ABC transporter, ATP-binding protein
MSMEG_3973		-3.4447	0.0000	N-methylhydantoinase
MSMEG_1712	*ytfQ*	-3.9384	0.0040	ABC transporter periplasmic-binding protein YtfQ
MSMEG_5941	*ksdD*	-3.9587	0.0229	3-oxosteroid 1-dehydrogenase
MSMEG_5894	*mam4A*	-4.2677	0.0061	Virulence factor Mce family protein
MSMEG_1115	*menG*	-4.4605	0.0271	Demethylmenaquinone methyltransferase

MSMEG_4517		-4.5240	0.0358	TetR-type transcriptional regulator of sulfur metabolism

Supplementary Table 2. Continued

Locus	Gene Name	Fold Change	p-value	Description
MSMEG_3139		-4.5733	0.0199	Enoyl-CoA hydratase/isomerase
MSMEG_0672		-4.9289	0.0288	GAF domain-containing protein
MSMEG_1771		-5.2604	0.0001	Methylase, putative
MSMEG_1392		-6.0292	0.0029	Alcohol dehydrogenase, class IV
MSMEG_4195		-6.9481	0.0000	Serine kinase
MSMEG_1097		-7.4238	0.0000	Glycosyl transferase, group 2 family protein
MSMEG_2436	*khpA*	-10.7928	0.0000	RNA-binding protein
MSMEG_1416		-53.0434	0.0000	Pyridine nucleotide-disulfide oxidoreductase
MSMEG_0854[a]				Two-component system sensor kinase
MSMEG_1743[a]				Fatty acid desaturase
MSMEG_2128[a]	*mbtK*			Lysine N-acyltransferase
MSMEG_2347[a]				Phytoene dehydrogenase
MSMEG_3787[a]				D-aminoacylase
MSMEG_6399[a]				Antigen 85-C
MSMEG_6767[a]				Mycocerosic acid synthase
MSMEG_6768[a]				Halogenase
MSMEG_2426[a]		-295.1716	8.16×10^{-08}	Nitrogen regulatory protein PII

a. Proteins only detected in wild-type strain
